# AgRP neurons trigger long-term potentiation and facilitate food seeking

**DOI:** 10.1038/s41398-020-01161-1

**Published:** 2021-01-05

**Authors:** Chunmei Wang, Wenjun Zhou, Yang He, Tiffany Yang, Pingwen Xu, Yongjie Yang, Xing Cai, Julia Wang, Hesong Liu, Meng Yu, Chen Liang, Tingting Yang, Hailan Liu, Makoto Fukuda, Qingchun Tong, Qi Wu, Zheng Sun, Yanlin He, Yong Xu

**Affiliations:** 1 Children’s Nutrition Research Center, Department of Pediatrics, Baylor College of Medicine, Houston, TX 77030 USA; 2 Brown Foundation Institute of Molecular Medicine, University of Texas Health Science Center at Houston, Houston, TX 77030 USA; 3 Department of Medicine, Division of Diabetes, Endocrinology and Metabolism, Baylor College of Medicine, Houston, TX 77030 USA; 4 Department of Molecular and Cellular Biology, Baylor College of Medicine, Houston, TX 77030 USA; 5 Pennington Biomedical Research Center, Louisiana State University System, Baton Rouge, LA 70808 USA

**Keywords:** Learning and memory, Physiology

## Abstract

Sufficient feeding is essential for animals’ survival, which requires a cognitive capability to facilitate food seeking, but the neurobiological processes regulating food seeking are not fully understood. Here we show that stimulation of agouti-related peptide-expressing (AgRP) neurons triggers a long-term depression (LTD) of spontaneous excitatory post-synaptic current (sEPSC) in adjacent pro-opiomelanocortin (POMC) neurons and in most of their distant synaptic targets, including neurons in the paraventricular nucleus of the thalamus (PVT). The AgRP-induced sEPCS LTD can be enhanced by fasting but blunted by satiety signals, e.g. leptin and insulin. Mice subjected to food-seeking tasks develop similar neural plasticity in AgRP-innervated PVT neurons. Further, ablation of the majority of AgRP neurons, or only a subset of AgRP neurons that project to the PVT, impairs animals’ ability to associate spatial and contextual cues with food availability during food seeking. A similar impairment can be also induced by optogenetic inhibition of the AgRP→PVT projections. Together, these results indicate that the AgRP→PVT circuit is necessary for food seeking.

## Introduction

Sufficient feeding is essential for survival. Hunger and/or hedonic properties of foods motivate animals and humans to eat, but the execution of feeding behavior requires more sophisticated skills, such as the cognitive capability to find food. While neurobiological processes that regulate motivations to eat have been extensively studied, the neural circuits that regulate cognitive functions to facilitate food-seeking remain to be fully understood.

Neurons that co-release agouti-related peptide (AgRP), neuropeptide Y (NPY), and GABA are located in the arcuate nucleus of the hypothalamus (ARH)^1,2^. These AgRP neurons play essential roles in promoting feeding behavior and ensure survival. For example, activation of AgRP neurons in satiated mice forces them to eat^3,4^. On the other hand, if AgRP neurons are ablated from adult mice, these mice starve to death within a few days^5,6^. AgRP neurons locally project to and synapse on adjacent pro-opiomelanocortin (POMC) neurons^7,8^, and provide GABAergic inputs to inhibit POMC neurons^9,10^. In addition, AgRP neurons also project to a number of long-distant targets throughout the brain^7,11–13^. These include the paraventricular nucleus of the hypothalamus (PVH), the paraventricular nucleus of the thalamus (PVT), the parabrachial nucleus (PBN), the bed nucleus of stria terminalis (BNST), the central amygdala (CeA), and the medial amygdala (MeA). Many of these projections (e.g. PVH, PVT, PBN, and BNST) have been shown to promote feeding when food is readily available^7,11,12^. Emerging evidence also indicates that some AgRP-originated circuits regulate more complex behaviors to facilitate feeding. For example, activation of AgRP neurons triggers foraging and repetitive behaviors, suppresses pain, and reduces anxiety^14–16^. Interestingly, stimulation of AgRP neurons in fed mice can drive cued food-seeking behavior in the presence of foot shock threats^17^. Further, the AgRP→MeA circuit was shown to suppress territorial aggression and reduce fear, which is implicated to facilitate food-seeking in the face of limited food^13^. AgRP neurons also regulate exploratory behavior, effects associated with AgRP projections to the reward circuitry^18^. Thus, AgRP-originated circuits appear to regulate multiple complex behaviors, and many of these behaviors are adaptive responses during starvation to facilitate food-seeking.

Neural plasticity is thought to be the neurobiological basis for information storage, and importantly both AgRP and POMC neurons exhibit neural plasticity in various conditions^19^. For example, excitatory and inhibitory synapses, as well as post-synaptic currents, in AgRP and POMC neurons are regulated by leptin signaling^20^. Other hormones, e.g. ghrelin^20–22^, estrogen^23^, and corticosterone^24^, can also regulate the neural plasticity of these ARH neurons. Interestingly, post-synaptic currents and excitability of AgRP and/or POMC neurons undergo dynamic changes at the fasting–feeding transition^25–28^, which also influences their responsiveness to leptin and ghrelin^29,30^. Various intracellular molecules, e.g. Sirt1^31^ and AMPK^22,25^, have been implicated to mediate changes in the neural plasticity of these neurons. Importantly, long-term potentiation or depression (LTP and LTD) can be triggered in both AgRP and POMC neurons upon stimulations of undefined pre-synaptic afferents, which can be influenced by animals’ feeding states^32^. However, whether AgRP neurons trigger LTP or LTD in POMC neurons or other targets via long-range projections remains unclear. Here, we detected the LTDs triggered by the activation of AgRP neurons. We further explored how these LTDs are regulated by hunger and metabolic signals, and delineated the underlying mechanisms. Finally, we examined the roles of AgRP neurons and their downstream circuits in facilitating food-seeking behavior.

## Results

### Stimulation of an AgRP neuron triggers sEPSC LTD in its downstream POMC neuron

**Fig. 1:**
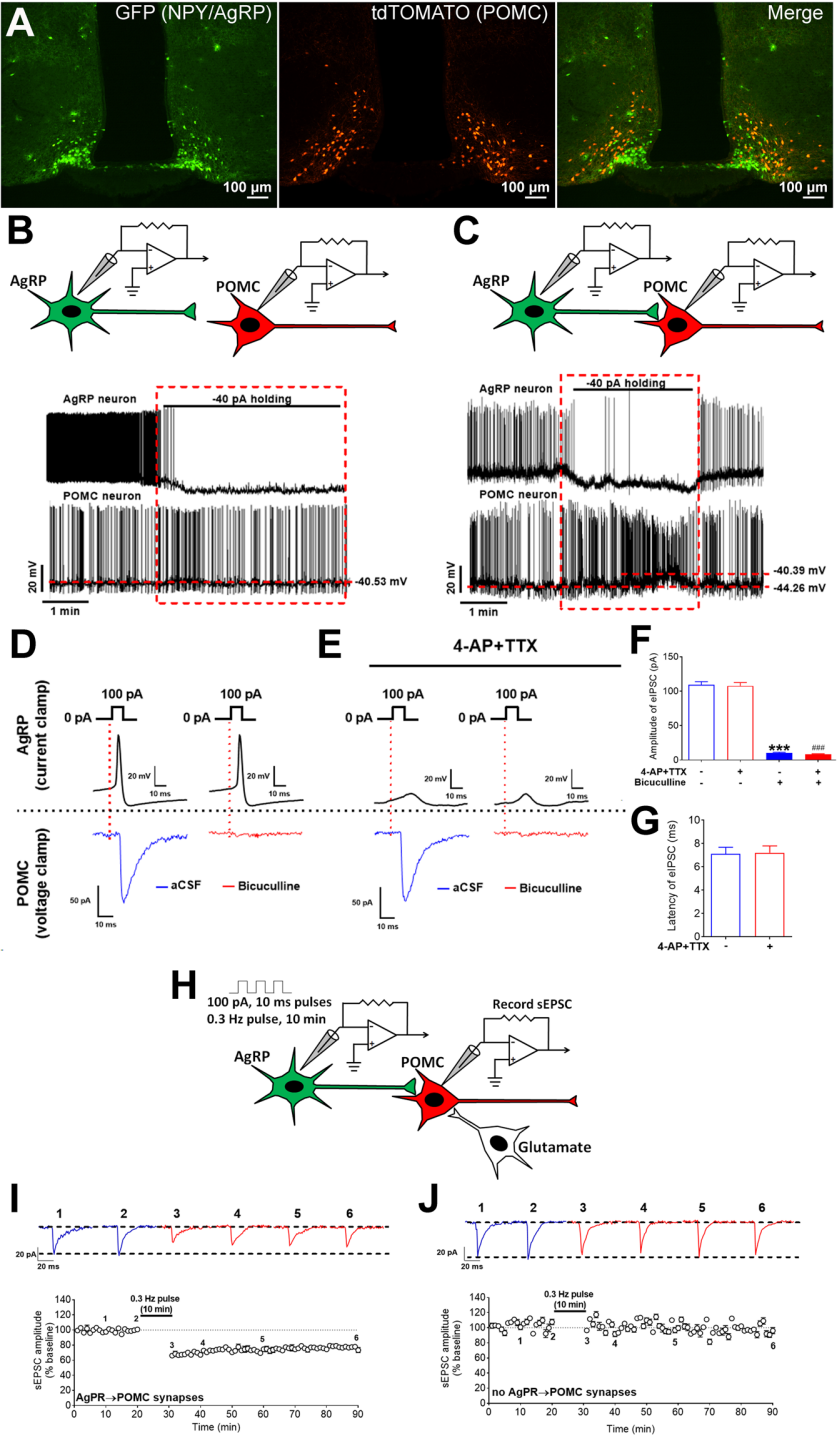
Stimulation of an AgRP neuron triggers sEPSC LTD in its downstream POMC neuron. **A** Representative microscopic images showing GFP-labelled AgRP neurons (left), tdTOMATO-labelled POMC neurons (middle), and merge (right) from NPY-GFP/POMC-CreERT2/Rosa26-LSL-tdTOMATO mice. **B**, **C** Representative action potential traces from an AgRP neuron and a POMC neuron that do not form a synapse (B) or from a pair that forms a synapse (**C**). **D**, **E** Representative traces for IPSCs recorded from a POMC neuron upon a positive current injected into its pre-synaptic AgRP neuron, in the absence (**D**) or the presence (**E**) of 4-AP and TTX. **F**, **G** Quantifications of IPSC amplitude (**F**) and latency (**G**). Data are mean ± SEM. *n* = 7 per group. ***P < 0.001 vs. 1st group; ###*P* < 0.001 vs. 2nd group in one-way ANOVA analyses followed by Tukey’s tests. **H** Schematic protocol to induce sEPSC LTD at the AgRP→POMC synapse. **I** Upper panel: representative sEPSC traces at the baseline and after AgRP neuron stimulation recorded from the POMC neuron that was synapsed by the AgRP neuron. Lower panel: quantitative changes in sEPSC amplitudes (as % of the baseline) in POMC neurons before and after the 10-min AgRP neuron stimulation. Data are mean ± SEM. *n* = 6 per group. **J** Upper panel: representative sEPSC traces at the baseline and after AgRP neuron stimulation recorded from the POMC neuron that was not synapsed by the AgRP neuron. Lower panel: quantitative changes in sEPSC amplitudes (as % of the baseline) in POMC neurons before and after the 10-min AgRP neuron stimulation. Data are mean ± SEM. *n* = 6 per group. Also see Fig. S1.

We first examined the well-established AgRP→POMC synapse within the ARH^7,33^. To this end, we crossed NPY-GFP^20^, POMC-CreERT2^34^, and Rosa26-LSL-tdTOMATO mouse alleles to generate NPY-GFP/POMC-CreERT2/Rosa26-LSL-tdTOMATO mice. Tamoxifen injection in these mice (0.2 g/kg, i.p., at 8 weeks of age) induced Cre activity and therefore led to expression of tdTOMATO selectively in mature POMC neurons; adjacent AgRP neurons were labelled by GFP (Fig. 1A). Brain slices containing the ARH were prepared from these mice (9 weeks, fed ad libitum), and double-patch whole-cell recordings were made in a pair of a randomly selected AgRP neuron (green) and an adjacent POMC neuron (red), respectively (Fig. S1A). In order to determine if the recorded AgRP neuron synapsed onto the recorded POMC neuron, we injected a negative current (–40 pA, 3–4 min) into the AgRP neuron and monitored the firing activity of POMC neurons under the current-clamp mode. In some pairs, inhibition of the AgRP neuron did not trigger obvious changes in firing of the recorded POMC neurons (Fig. 1B), suggesting that this pair did not form an AgRP→POMC synapse. In other pairs, we observed instant elevations of firing activity in the POMC neuron upon AgRP neuron inhibition, which instantly recovered upon the completion of the negative currents injected into the AgRP neuron (Fig. 1C), suggesting that this pair likely formed an AgRP→POMC synapse. To further confirm the formation of the AgRP→POMC synapse in these AgRP/POMC pairs, we applied a positive current (100 pA, 10 ms pulse) onto the AgRP neuron (under the current-clamp mode), and detected strong evoked inhibitory post-synaptic currents (eIPSC) in the POMC neuron under the voltage-clamp mode (Fig. 1D). The eIPSC in POMC neurons was blocked by bath perfusion of 50 µM bicuculline (GABA_A_ receptor antagonist, Fig. 1D, F). Importantly, the amplitude and latency of the evoked eIPSC were not affected by bath perfusion of 400 µM 4-AP and 1 µM TTX (Fig. 1E–G), further confirming the monosynaptic connection between the upstream AgRP neuron and the downstream POMC neuron.

For each recorded pair that formed an AgRP→POMC synapse, we observed an LTD of spontaneous excitatory post-synaptic current (sEPSC) in POMC neurons upon a train stimulation of AgRP neurons. Briefly, we injected a train of positive currents (100 pA, 10 ms pulses at 0.3 Hz for 10 min) into the pre-synaptic AgRP neuron, and sEPSC in the post-synaptic POMC neuron was recorded for 20 min (0–20 min) prior to the train stimulation and for 60 min (30–90 min) afterward (Fig. 1H). Strikingly, we observed that the amplitude of sEPSC in POMC neurons was robustly reduced (to 74% of the baseline, Fig. 1I), while no changes in sEPSC frequency were noted (data not shown). In other words, activation of the AgRP neuron has a long-term effect to suppress the responsiveness of the downstream POMC neuron to excitatory synaptic inputs (likely via glutamate). As a negative control, we also tested the same sEPSC LTD protocol in AgRP-POMC pairs that did not form synapses (see Fig. 1B), and we failed to detect any LTD (Fig. 1J).

### AgRP→POMC sEPSC LTD is regulated by nutritional and metabolic signals

**Fig. 2:**
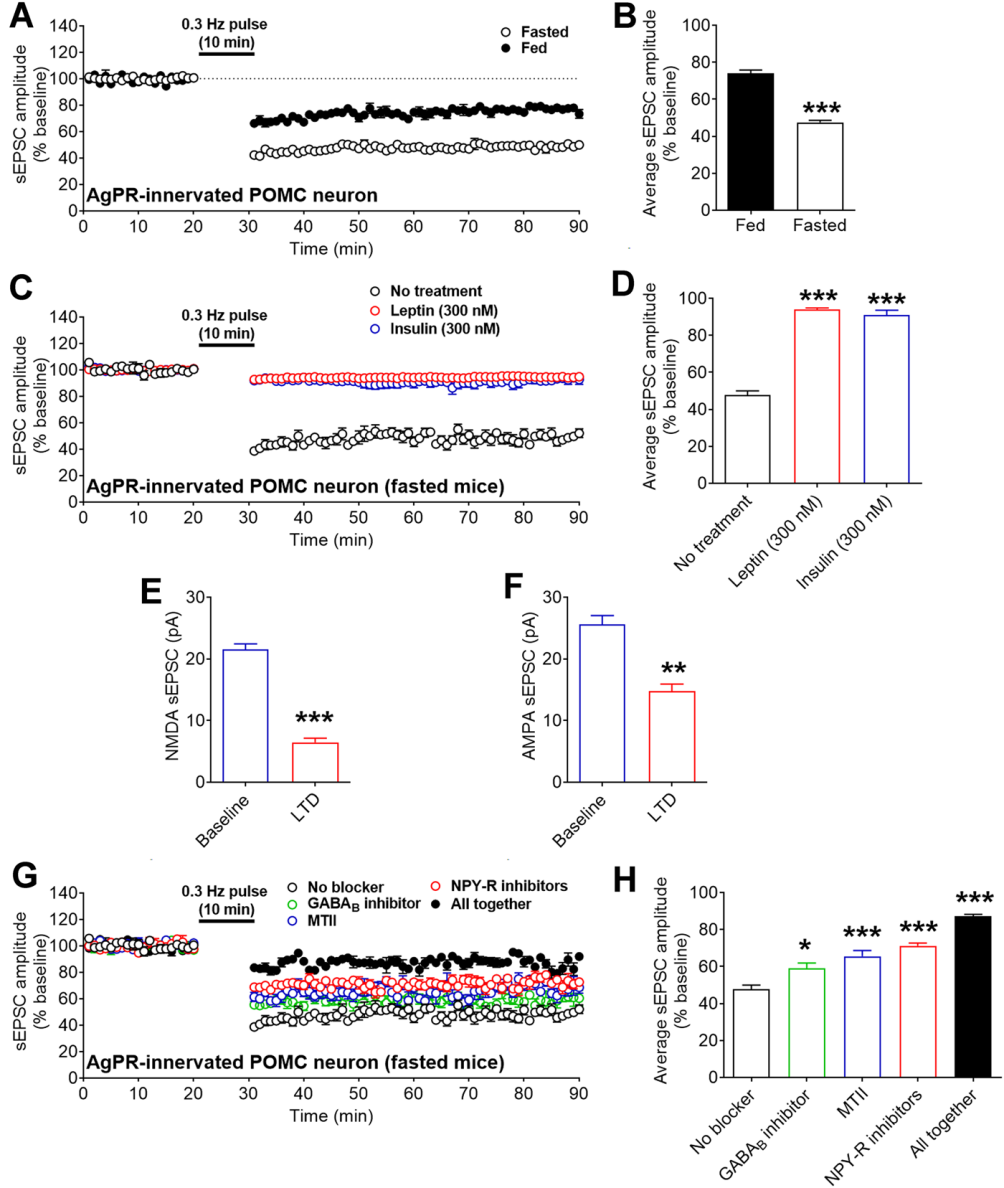
Characterization of the sEPSC LTD in AgRP-innervated POMC neurons. **A** Temporal changes in sEPSC amplitudes in AgRP-innervated POMC neurons from mice that were fed ad libitum or fasted for 24 h. **B** The average sEPSC amplitude after AgRP stimulation in (**A**). Data are mean ± SEM. *n* = 6 per group. ***P < 0.001 in two-tailed unpaired t tests. **C** Temporal changes in sEPSC amplitudes in AgRP-innervated POMC neurons (from fasted mice) after the brain slices were pre-incubated with vehicle, leptin, or insulin. **D** The average sEPSC amplitude after AgRP stimulation in (**C**). Data are mean ± SEM. *n* = 5 or 6 per group. ***P < 0.001 vs. the no treatment group in one-way ANOVA analyses followed by post hoc Tukey’s tests. **E**, **F** Amplitudes of NMDA (**E**) and AMPA (**F**) sEPSC in POMC neurons before (baseline) and after (LTD) the 10-min AgRP neuron stimulation. Data are mean ± SEM. *n* = 6 per group. **P < 0.01 and ***P < 0.001 in two-tailed paired t tests. **G** Temporal changes in sEPSC amplitudes in POMC neurons before and after the 10-min AgRP neuron stimulation (from fasted mice) after the brain slices were pre-incubated with various blockers. **H** The average sEPSC amplitude after AgRP stimulation in (**G**). Data are mean ± SEM. *n* = 6 or 7 per group. **P < 0.01 and ***P < 0.001 vs. the no blocker group in one-way ANOVA analyses followed by post hoc Tukey’s tests. Also see Fig. S1.

We then compared the AgRP→POMC sEPSC LTD in mice that were fed ad libitum or fasted for 24 h, and found that the fasting significantly enhanced the LTD (from 74 to 47% of the baseline, Fig. 2A, B), indicating that this neuroplasticity is strengthened by hunger. Further, we incubated ARH-containing hypothalamic slices from fasted mice with 300 nM leptin for 1 h, and found that the leptin treatment significantly attenuated the AgRP→POMC sEPSC LTD (Fig. 2C, D). Similarly, incubation with insulin (300 nM for 1 h) also drastically suppressed the AgRP→POMC sEPSC LTD (Fig. 2C, D). Together, these data indicate that the sEPSC LTD in AgRP-innervated POMC neurons can be enhanced by food deprivation and be attenuated by satiety signals (e.g. leptin and insulin).

### Mechanisms for the AgRP→POMC sEPSC LTD

We next sought to explore the molecular mechanism underlying the sEPSC LTD in AgRP-innervated POMC neurons. First, we found that the amplitudes of both NMDA and AMPA sEPSC in POMC neurons were significantly reduced during the LTD phase (after the train stimulation of AgRP neurons) compared to the baseline levels (Figs. 2E, F and S1B), indicating that reduced NMDA and AMPA neurotransmissions both contributed to the development of the sEPSC LTD in AgRP-innervated POMC neurons. We then assessed the roles of the three neuro-messages released from pre-synaptic AgRP neurons, namely GABA, AgRP, and NPY. Notably, the GABA_A_ receptor inhibitor (bicuculline) was added into the preparation for the sEPSC LTD recording protocol at all times; therefore, the formation of the sEPSC LTD did not require GABA_A_ receptor-mediated signals in our experimental setting. We then tested the functional relevance of the GABA_B_ receptor and found that pre-treatment with a GABA_B_ receptor inhibitor, CGP52432 (50 µM), induced a small but significant reduction of sEPSC LTD in fasted mice (Fig. 2G, H). Since AgRP functions as an endogenous antagonist of the melanocortin 3 or 4 receptors (MC3R or MC4R)^35^, we used the MC3/4R agonist, MTII (100 µM), to negate the effects of AgRP. Interestingly, MTII significantly attenuated the sEPSC LTD (from 47 to 66% of the baseline, Fig. 2G, H). It has been reported that POMC neurons abundantly express 3 NPY receptors, namely NPY1R, NPY2R, and NPY5R^36–38^. Thus, we used a cocktail of BVD10 (50 µM, an NPY1R antagonist), JNJ5207787 (100 µM, an NPY2R antagonist), and NPY5RA972 (100 µM, an NPY5R antagonist), to block NPY actions. Remarkably, these NPY receptor inhibitors significantly attenuated the sEPSC LTD (from 47 to 71% of the baseline, Fig. 2G, H). Finally, a combination of all these inhibitors produced an even bigger attenuation in the sEPSC LTD (from 47 to 87% of the baseline, Fig. 2G, H). Thus, these results indicate that all three neuro-messages released from AgRP neurons additively contribute to the formation of the sEPSC LTD in AgRP-innervated POMC neurons, with more robust effects resulting from AgRP and NPY than those from GABA.

### AgRP neurons trigger sEPSC LTDs in distant synaptic targets

**Fig. 3:**
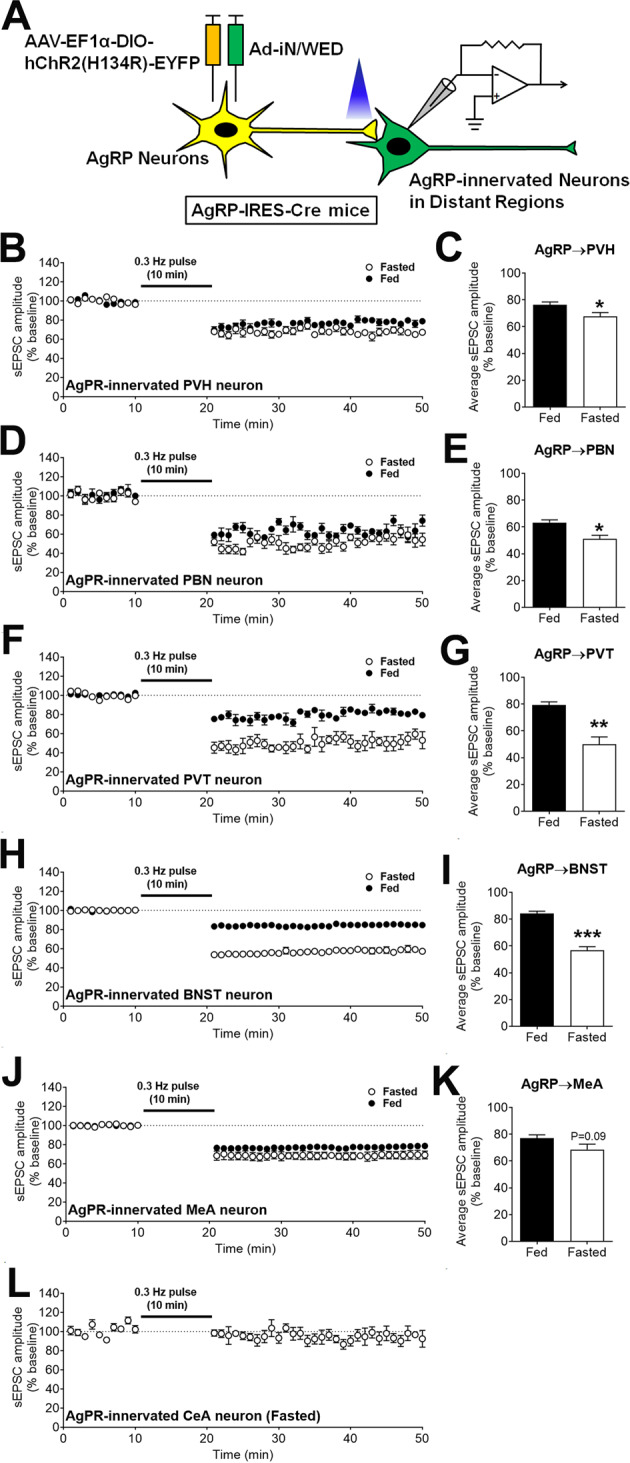
AgRP neurons trigger sEPSC LTDs in distant synaptic targets. **A** Schematic illustration to identify distant AgRP-originated synapses. **B**, **D**, **F**, **H**, **J**, **L** Temporal changes in sEPSC amplitudes before and after the 10-min photostimulation in AgRP-innervated PVH (**B**), PBN (**D**), PVT (**F**), BNST (**H**), MeA (**J**), CeA (**L**) neurons from mice that were fed ad libitum or fasted for 24 h. **C**, **E**, **G**, **I**, **K** The average sEPSC amplitude after AgRP stimulation in AgRP-innervated neurons in (**B**, **D**, **F**, **H**, **J**), respectively. Data are mean ± SEM. *n* = 4, 5, 6, or 7 per group. *P < 0.05, **P < 0.01, and ***P < 0.001 vs. fed group in two-tailed unpaired t tests. Also see Figs. S2 and S3.

In addition to the adjacent POMC neurons, AgRP neurons also project to and synapse on neurons in several other distant brain regions, including the PVH, PVT, PBN, BNST, CeA^11^, and MeA^13^. Thus, we further tested whether stimulation of AgRP neurons can trigger similar sEPSC LTDs in these distant synapses. To this end, we stereotaxically injected AAV-EF1α-DIO-hChR2(H134R)-EYFP and Ad-iN/WED viruses into the ARH of AgRP-IRES-Cre mice. AAV-EF1α-DIO-hChR2(H134R)-EYFP virus expressed channelrhdopsin-2 (ChR2) selectively in AgRP cell bodies and fibers/terminals, which enabled photostimulation (Fig. 3A). The Ad-iN/WED virus^39^ expressed GFP-tagged wheat germ agglutinin (WGA-GFP) in a Cre-dependent manner and therefore only filled the AgRP neurons. Since WGA-GFP can anterogradely pass the synapse, it labelled the downstream neurons that were innervated by AgRP terminals. Thus, GFP-labelled neurons in distant brain regions were likely the synaptic targets of AgRP neurons (Figs. 3A and S2A, B). To confirm the synaptic connectivity between the ChR2-labelled AgRP terminals and WGA-labelled neurons, we first used the ChR2-assisted circuit mapping (CRACM) protocol^40^. Thus, for each WGA-labelled neuron, we detected strong time-locked eIPSCs upon application of blue light pulses (473 nM, 40 mW, 10 ms/pulse) onto the ChR2-labelled fibers/terminals. Importantly, these eIPSCs persisted in the presence of 400 µM 4-AP and 1 µM TTX but were blocked by 50 µM bicuculline (Fig. S2B–M). These data confirmed that the WGA-labelled neurons were monosynaptic targets of AgRP neurons. Once we confirmed the synaptic connectivity, we then applied a train of blue light pulses (473 nM, 40 mW, 0.3 Hz pulses for 10 min) to the ChR2-labelled fibers/terminals, and sEPSC in the WGA-labelled neuron were recorded for 10 min (0–10 min) prior to the train photostimulation and for 30 min (20–50 min) afterward. Similarly, as we observed in the AgRP-innervated POMC neurons, we detected sEPSC LTDs (as demonstrated by prolonged reductions in sEPSC amplitude) in the WGA-labelled neurons in the PVH, PBN, PVT, BNST, and MeA (Fig. 3B–K). In addition, the amplitudes of both NMDA and AMPA sEPSC in all these target neurons were significantly reduced during the LTD phase (after the train stimulation of AgRP neurons) compared to the baseline levels (Fig. S3A–J). Interestingly, the sEPSC LTD at most of these targeted regions was significantly enhanced by fasting, although the LTD in AgRP-innervated MeA neurons was not significantly affected by the feeding conditions (Fig. 3B–K). Notably, no sEPSC LTD was detected in the AgRP-innervated CeA neurons even in fasted mice (Fig. 3L and S3K, L). We further examined the contributions of GABA, AgRP, and NPY to the sEPSC LTD in PVT neurons, and found that only AgRP and NPY actions are required but GABA signals play a minimal role in the formation of the sEPSC LTD in AgRP-innervated PVT neurons (Fig. S3M, N). In summary, these results indicate that prolonged activation of AgRP neurons triggers sEPSC LTDs in most of their distant synaptic targets.

### Loss of AgRP neurons impairs the food-seeking behavior

Since the synaptic plasticity is thought to be the neurobiological basis for information storage, the sEPSC LTDs observed at multiple AgRP-innervated regions led us to hypothesize that AgRP neurons are required for cognitive functions in mice. To test this possibility, we injected diphtheria toxin (DT, 50 ng/g, s.c.) into AgRP^DTR/+^ mice^6^ during the first week after birth. As reported previously^6^, this resulted in specific ablation of the majority of AgRP neurons in the ARH (AgRP^neoAblation^ mice), as validated by 85–87% loss of AgRP and NPY mRNAs but normal POMC mRNAs in these AgRP^neoAblation^ mice compared to control mice (Fig. S4A).

**Fig. 4:**
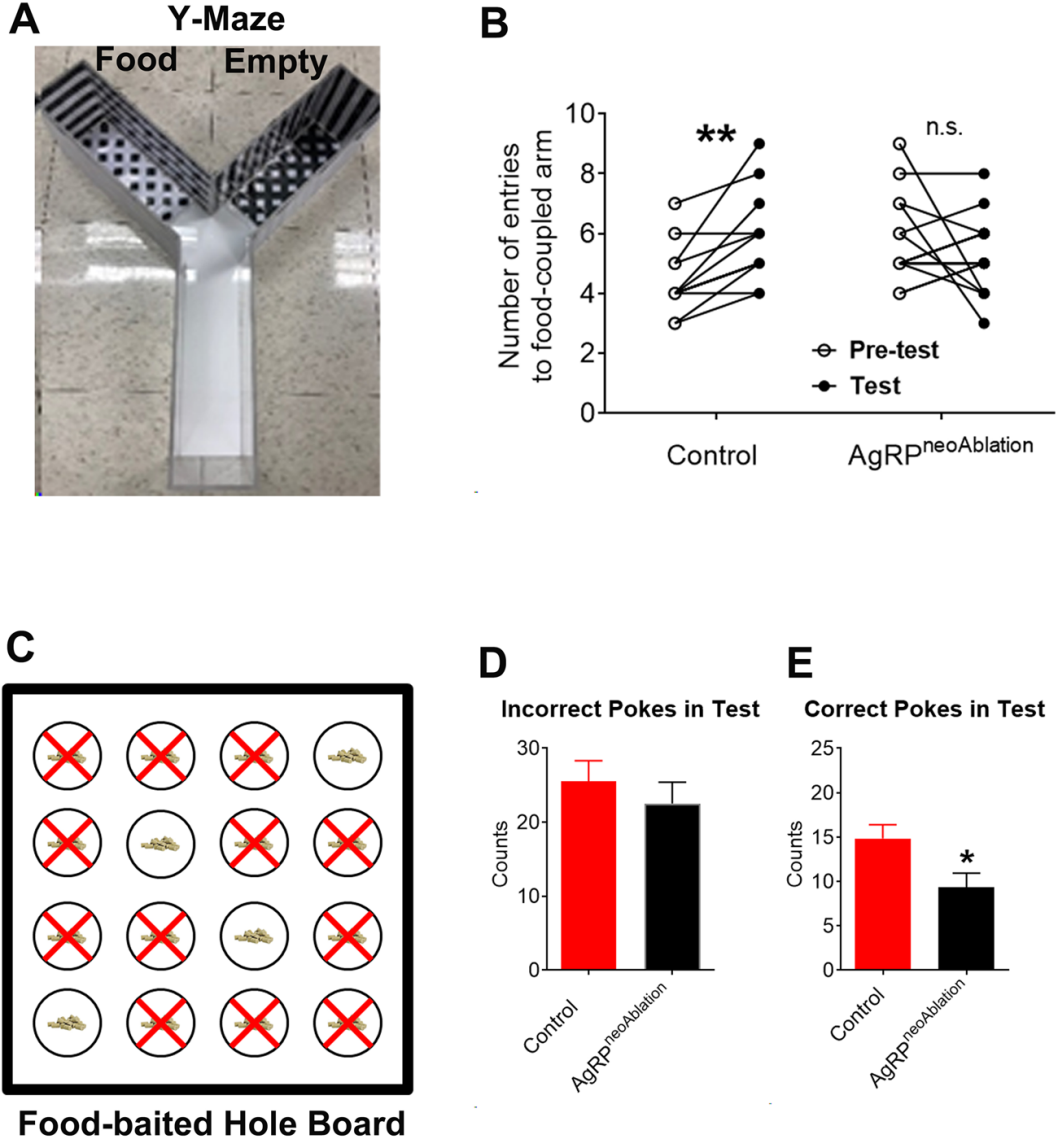
Loss of AgRP neurons impairs food seeking. **A** A photo of the Y-maze. **B** Number of entries to food-coupled arm by control and AgRP^neoAblation^ mice during pre-test and test in the Y-maze assay. *n* = 14 or 15 per group. **P < 0.01 between pre-test and test in two-way ANOVA repeated measurement followed by post-hoc-Sidak tests. Note that the number of data points may appear fewer than the *n* values because multiple mice showed the same number of entries during the pre-test or test. **C** Schematic presentation of the hole-board with food baits. **D**, **E** Incorrect (**D**) and correct pokes (**E**) of control and AgRP^neoAblation^ mice in the food-baited hole board test. Data are mean ± SEM. *n* = 16 or 17 per group. *P < 0.05 vs. control in two-tailed unpaired t tests. Also see Fig. S4.

It has been recently reported that activation of AgRP neurons, either by food deprivation or by ChR2-mediated optogenetic stimulation, enhances mouse’s learning for spatial cues associated with food pellets in a Y-maze test^15^. Thus, we first tested the cognitive functions of AgRP^neoAblation^ mice using the Y-maze test. Mice were restricted to 85% of their initial body weight to enhance motivation for food. These mice were subjected to five conditioning sessions, in which they were trained to associate one arm of the maze with a 1 g food pellet utilizing visual and spatial cues (Fig. 4A). Animals’ choice between the two arms of the maze was tested before (pre-test) and after (test) these conditioning sessions. As expected, control mice displayed a significant increase in entering the food-coupled arm in the test compared to the pre-test (Fig. 4B), indicating that they learned and remembered the cues associated with food availability. On the other hand, AgRP^neoAblation^ mice showed comparable performance in the pre-test vs. the test (Fig. 4B), indicating that these mice failed to remember cues associated with food availability.

We then tested whether AgRP^neoAblation^ mice can learn and remember spatial cues associated with food availability using a modified food-baited hole board test^41^ comprised of four training sessions followed by a test session (after an overnight fasting). In each session, mice were allowed 10 min to explore a 16-hole board apparatus (50 cm × 50 cm in size), in which four holes had an accessible food pellet in each and the rest holes had inaccessible food pellets; the food-accessible hole remained the same throughout the five sessions (Fig. 4C). During the test session, we found that food-deprived control mice and AgRP^neoAblation^ mice showed comparable nose pokes to the food-inaccessible holes (incorrect pokes, Fig. 4D), but AgRP^neoAblation^ mice showed significantly fewer nose pokes to the food-accessible holes (correct pokes) than controls (Fig. 4E). Notably, these parameters were all comparable between control and AgRP^neoAblation^ mice during the initial training session (Fig. S4B, C). Thus, these results indicate that AgRP^neoAblation^ mice have an impaired ability to associate spatial/contextual cues with food availability, which is required for food seeking. In addition, AgRP^neoAblation^ mice showed normal food intake compared to their controls when ad libitum fed with chow (Fig. S4D). Notably, after a 24 h fasting, the refeeding response was also comparable between AgRP^neoAblation^ and control mice (Fig. S4D). The lack of food intake phenotype in our AgRP^neoAblation^ mice is not consistent with a recent report^42^, which presumably reflects the incomplete ablation of AgRP neurons (Fig. S4A). Nevertheless, the impaired performance observed in the Y-maze test and in the food-baited hole board test was not likely due to impaired hunger sensing.

We further examined whether AgRP^neoAblation^ mice had deficits in other learning/memory tasks not involving foods as bait or motivation. In the Morris water maze (MWM) tests, AgRP^neoAblation^ mice showed normal learning curves compared to controls (Fig. S4E). In the fear conditioning test, AgRP^neoAblation^ mice and control mice showed comparable freeze behaviors in the context in which they had received a foot shock (Fig. S4F), and in response to the cue tone previously associated with the foot shock (Fig. S4G), suggesting that the fear memories were intact in AgRP^neoAblation^ mice.

### Y-maze conditioning induced neural plasticity in AgRP-innervated PVT neurons

**Fig. 5:**
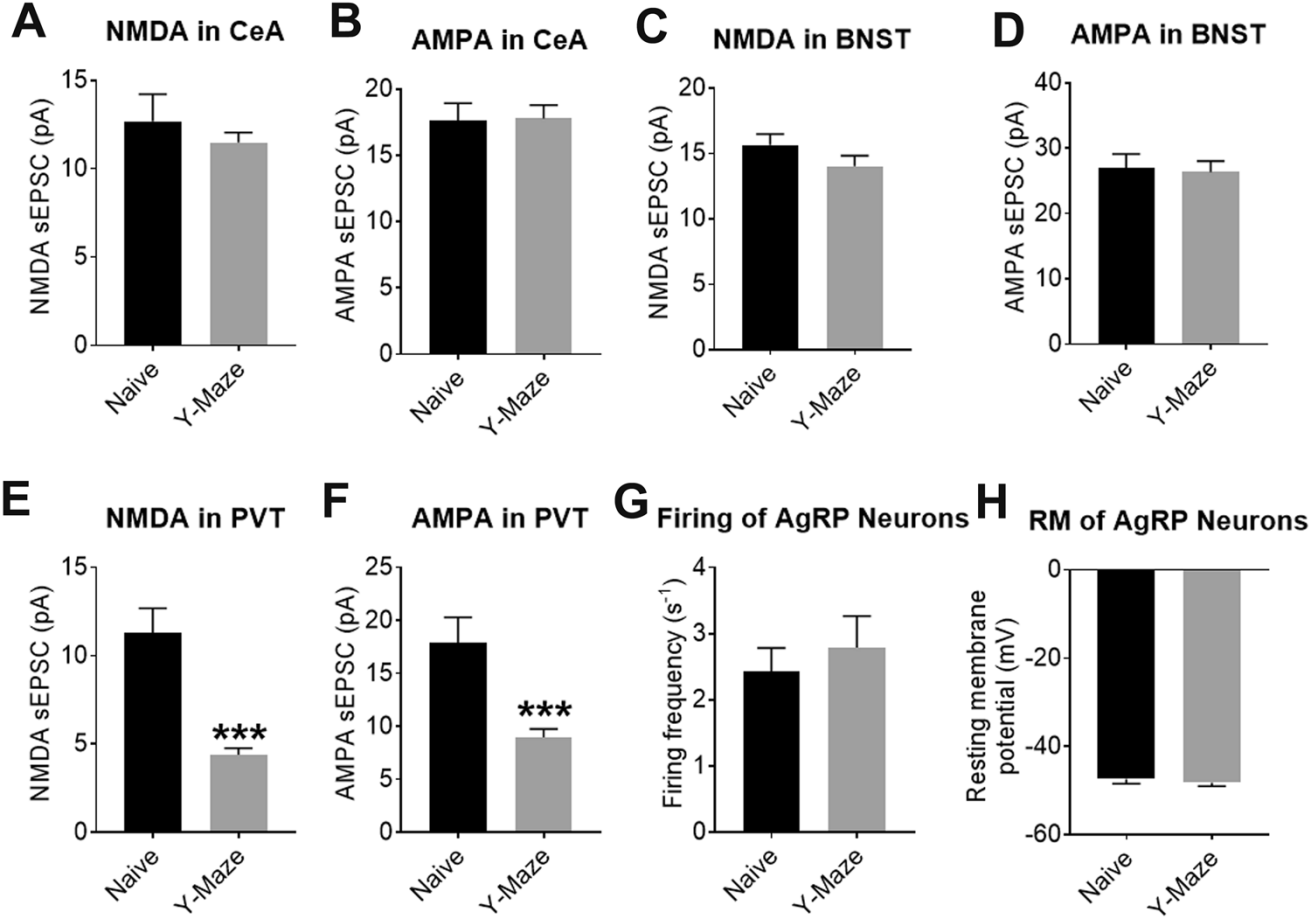
Y-maze conditioning induced neural plasticity in AgRP-innervated PVT neurons. **A**, **C**, **E** NMDA sEPSC in the AgRP-innervated neurons in the CeA (**A**), BNST (**C**), and PVT (**E**) from naive mice or Y-maze-trained mice. **B**, **D**, **F** AMPA sEPSC in the AgRP-innervated neurons in the CeA (**B**), BNST (**D**), and PVT (**F**) from naive mice or Y-maze-trained mice. Data are mean ± SEM. *n* = 5 or 10 per group. ***P < 0.001 vs. naive group in two-tailed unpaired t tests. **G**, **H** Firing frequency (**G**) and resting membrane potential (**H**) of AgRP neurons from naive mice or Y-maze-trained mice. Data are mean ± SEM. *n* = 12 per group. Also see Fig. S5.

We then asked which AgRP-originated circuits may facilitate learning/memory for cues associated with food availability during food seeking. Among the AgRP target regions, the CeA^43^, the BNST^44^, and the PVT^45^ have been implicated in the regulations of cognitive functions. Thus, we surveyed whether neural plasticity develops at these AgRP-innervated neurons during the Y-maze test. To this end, we injected AAV-EF1α-DIO-hChR2(H134R)-EYFP and Ad-iN/WED viruses into the ARH of AgRP-IRES-Cre mice. After recovery, these mice were subjected to the Y-maze conditioning for 4 days as described above; naive mice were used as controls. After these mice were refed ad libitum for 24 h, we examined the NMDA and AMPA sEPSC in AgRP-targeted neurons in the CeA, BNST, and PVT. As described above, WGA-labelled neurons in each region were first located as putative AgRP-innervated neurons, and then further confirmed by the existence of time-locked IPSCs evoked by photostimulation of ChR2-expressing AgRP terminals. Among these AgRP-innervated neurons, we found that Y-maze conditioning did not induce significant changes in NMDA or AMPA sEPSC of those in the CeA or in the BNST (Fig. 5A–D). Interestingly, compared to control mice, mice trained in the Y-maze showed robust reductions in NMDA and AMPA sEPSC of AgRP-innervated neurons in the PVT (Fig. 5E, F and S5). Meanwhile, the firing frequency and resting membrane potential (RM) of AgRP neurons in Y-maze-trained mice were comparable to those from control mice (Fig. 5G, H). Thus, the reduced NMDA and AMPA sEPSC observed in AgRP-innervated PVT neurons were likely due to the development of neural plasticity during the Y-maze conditioning, rather than to increased AgRP neuron activity at the time of recording. In addition, since reductions in NMDA and AMPA sEPSC were associated with and likely contributed to the sEPSC LTDs that we detected in slice recordings (Figs. S3E, F), we suggest that sEPSC LTDs developed in the AgRP-innervated PVT neurons in vivo after the Y-maze conditioning.

### The AgRP→PVT circuit facilitates the food-seeking behavior

**Fig. 6:**
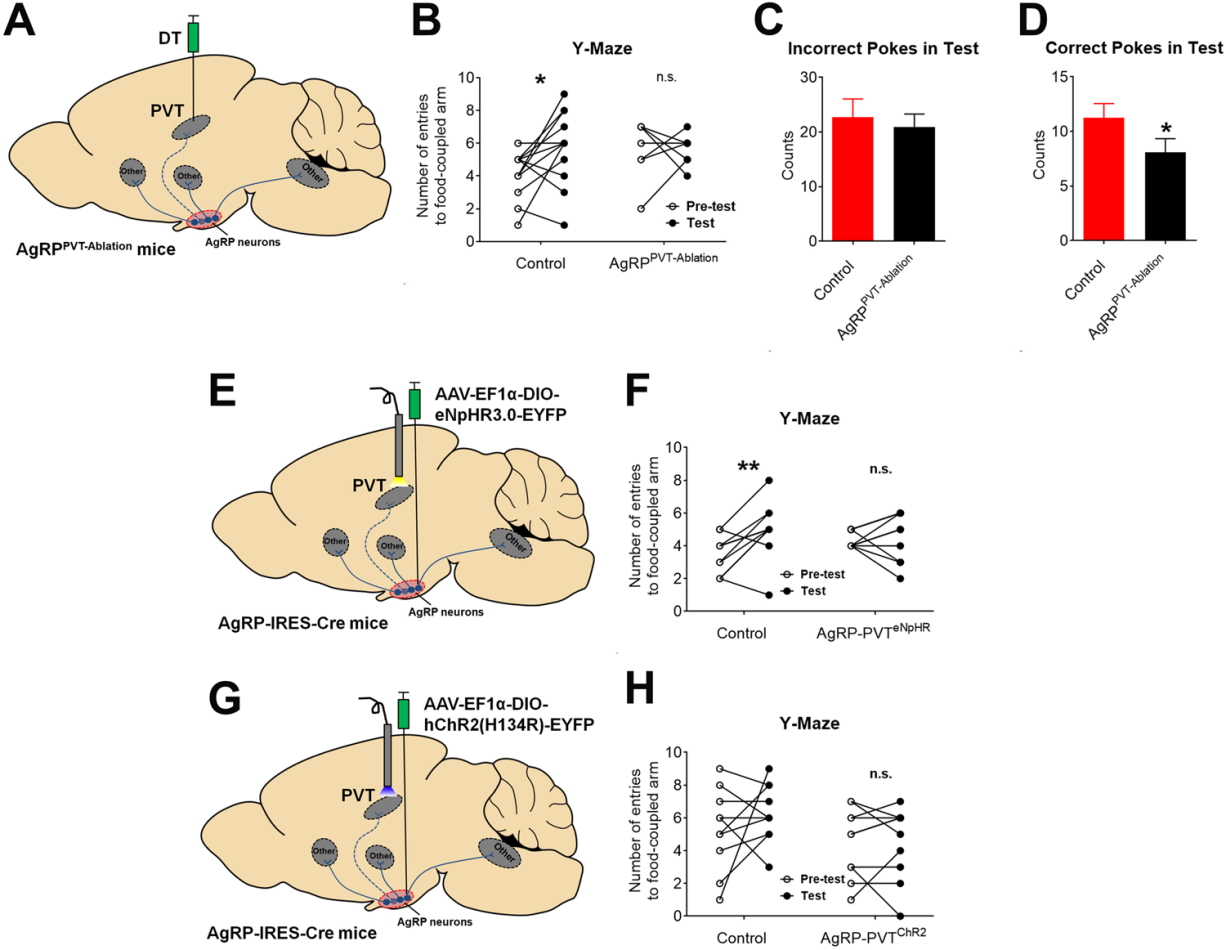
The AgRP→ PVT circuit regulates food seeking. **A** Schematic illustration to selectively ablate AgRP neurons projecting to the PVT. **B** Number of entries to food-coupled arm by control and AgRP^PVT-Ablation^ mice during pre-test and the test in the Y-maze assay. *n* = 7 or 12 per group. *P < 0.05 between pre-test and the test in two-way ANOVA repeated measurement followed by post hoc Sidak tests. Note that the number of data points may appear fewer than the *n* values because multiple mice showed the same number of entries during the pre-test or the test. **C**, **D** Incorrect (**C**) and correct pokes (**D**) of control and AgRP^PVT-Ablation^ mice in the food-baited hole board test. Data are mean ± SEM. *n* = 12 or 15 per group. *P < 0.05 vs. control in one-tailed unpaired t tests. **E** Schematic illustration to selectively inhibit AgRP→PVT projections. **F** Number of entries to the food-coupled arm in the pre-test and the test in the Y-maze assay by control mice and mice with inhibited AgRP→PVT projections. *n* = 10 per group. *P < 0.05 between the pre-test and the test in two-way ANOVA repeated measurement followed by post hoc Sidak tests. Note that the number of data points may appear fewer than the *n* values in because multiple mice showed the same number of entries during the pre-test or the test. **G** Schematic illustration to selectively activate AgRP→PVT projections. **H** Number of entries to the food-coupled arm in the pre-test and the test in the Y-maze assay by control mice and mice with activated AgRP→PVT projections. *n* = 10 per group. Note that the number of data points may appear fewer than the *n* values in because multiple mice showed the same number of entries during the pre-test or the test. Also see Figs. S6, S7.

The neural plasticity in AgRP-innervated PVT neurons we observed in Y-maze-trained mice suggests that this neural circuit may facilitate food seeking during the Y-maze task. To test this possibility, we stereotaxically injected DT (1 ng in 200 nl saline) into the PVT of AgRP^DTR/+^ mice to selectively ablate AgRP neurons that project to the PVT (Fig. 6A). Post hoc analyses counting the AgRP neurons in the ARH (GFP-labelled neurons by an NPY-GFP allele) revealed that DT-injected mice lose a subset of AgRP neurons in the anterior portion of the ARH compared to control mice (Figs. S6A, B). We further validated the loss of AgRP-positive fibers/boutons in the PVT, but not in the PVH (Figs. S6C, D). Similar to AgRP^neoAblation^ mice, AgRP^PVT-Ablation^ mice consumed comparable amount of chow diet as their controls, regardless of when they were fed ad libitum or refed after a 24 h fast (Fig. S6E). In the Y-maze test, these AgRP^PVT-Ablation^ mice showed a comparable performance in the pre-test vs. the test, while control mice displayed a significant increase in entering the food-coupled arm in the test compared to the pre-test (Fig. 6B). After training in the food-baited hole board test, AgRP^PVT-Ablation^ mice showed significantly fewer correct pokes than controls, although the number of incorrect pokes was comparable between the two groups (Fig. 6C, D). Importantly, these parameters were comparable between control and AgRP^PVT-Ablation^ mice during the initial training session (Figs. S6F, G). In addition, AgRP^PVT-Ablation^ mice showed normal performances as control mice in the fear conditioning test (Figs. S6H, I).

To further support the role of the AgRP→PVT circuit in the food-seeking behavior, we stereotaxically injected AAV-EF1α-DIO-eNpHR3.0-EYFP into the ARH of AgRP-IRES-Cre mice and implanted an optic fiber to target the PVT (Figs. 6E and S7A–D). These mice and their controls were then calorie-restricted and subjected to the Y-maze conditioning sessions, during which yellow light pulses were applied to the PVT to selectively inhibit the AgRP→PVT projections. While control mice displayed a significant increase in entering the food-coupled arm in the test compared to the pre-test, such preference was not detected in mice with the AgRP→PVT projections inhibited during the conditioning (Fig. 6F). We then tested whether activation of the AgRP→PVT circuit in mice fed ad libitum can enhance their capability to seek food. To this end, we stereotaxically injected AAV-EF1α-DIO-hChR2(H134R)-EYFP into the ARH of AgRP-IRES-Cre mice and implanted an optic fiber to target the PVT (Figs. 6G and S7E–G). These mice and their controls were fed ad libitum and subjected to the Y-maze conditioning sessions, during which blue light pulses were applied to the PVT to selectively activate the AgRP→PVT projections. However, optogenetic stimulation of the AgRP→PVT circuit had no effect on the Y-maze performance (Fig. 6H). Together, these data indicate that the AgRP→PVT circuit is required to facilitate food seeking by associating spatial/contextual cues with food availability in hungry mice, but activation of this circuit alone in satiated mice is not sufficient for this function.

## Discussion

Here we used a unique tri-transgenic mouse model, which allowed us to visualize both AgRP neurons and the adjacent POMC neurons in the ARH. The double-patch approach was used to record a single AgRP neuron and a single POMC neuron that formed an AgRP→POMC synapse. Notably, the randomly selected AgRP-POMC pairs may not always form a synapse. Thus, we used two criteria to confirm the synaptic connectivity between the two neurons. One was the instant activation of POMC neuron upon AgRP neuron inhibition; the other was the time-locked IPSCs in the POMC neuron evoked by activation of the AgRP neuron, a response that was blocked by bicuculline but not by 4-AP + TTX. These responses are consistent with the well-established local circuit that AgRP neurons synapse on and inhibit POMC neurons^7–10,33^. At these confirmed AgRP→POMC synapses, we detected prolonged reductions in sEPSC in the POMC neuron after a short stimulation of the AgRP neuron, which confirmed the existence of the sEPSC LTD. Importantly, within the AgRP-POMC pairs that did not form synapses, we failed to observe such LTDs. We further extended these observations to other distant target neurons innervated by AgRP neurons by combining the WGA trans-synaptic anterograde tracer and ChR2-assisted circuit stimulation. Since WGA may also travel retrogradely^46^, the WGA-labelled neurons in the known AgRP-downstream regions may not be the synaptic targets of AgRP neurons. To further establish the synaptic connectivity between AgRP neurons and these WGA-labelled neurons, we used the CRACM protocol to confirm the existence of time-locked IPSCs in WGA-labelled neurons upon photostimulation of the AgRP terminals. Interestingly, we detected similar LTDs in most of these long-distant targets, e.g. the PVH, PBN, PVT, BNST, and MeA. Together, these results revealed the existence of neural plasticity in many AgRP-innervated neurons.

The LTDs in all these AgRP-innervated neurons were associated with reductions in both NMDA and AMPA-mediated sEPSC. Given the rapid development of the LTDs, it is unlikely that expression levels of NMDA and/or AMPA receptor subunits are altered. Rather, we suggest that the 10-min AgRP neuron stimulation may trigger endocytosis of the glutamatergic receptor subunits, which is a common molecular mechanism for the formation of LTDs at other synapses^47^. However, it is worth noting that AgRP neurons per se do not secrete glutamate^48^. Thus, the LTDs detected in the AgRP-innervated neurons were heterosynaptic in nature^49,50^. In other words, neuro-messages released from the AgRP neuron act upon its target neuron (e.g. a POMC neuron) to suppress the target neuron’s responsiveness to glutamatergic inputs (coming from a third neuron). Such heterosynaptic LTDs have been reported primarily within the hippocampal synapses^49,50^, and play important roles in a variety of neural processes including associative learning and homeostasis of synaptic input^51^. We further demonstrated that AgRP and NPY both contribute substantially to the formation of the LTDs in POMC neurons, while GABA, via GABA_B_ receptors, is also involved to a lesser extent. Interestingly, in the AgRP-innervated PVT neurons, only AgRP and NPY actions are required for the formation of the sEPSC LTDs, while GABA_B_-mediated signals are not. It is worth mentioning that due to the addition of bicuculline in our slice preparations to allow measurements of sEPSC, we were not able to experimentally examine the contributions of GABA via GABA_A_ receptors. Notably, the LTDs at most of these regions (except for the MeA) were enhanced by hunger; on the other hand, the satiety signals (leptin and insulin) largely attenuated the LTDs in POMC neurons innervated by AgRP neurons. It is known that food deprivation elevates AgRP and NPY transcriptions^52^. Thus, we suggest that the regulations of the LTDs by hunger are at least partly mediated by alterations in the expression of these key neuropeptides in AgRP neurons. Although leptin and insulin have been reported to decrease the expression of AgRP and NPY^53,54^, the 1-h incubation of these hormones in our studies may not result in sufficient reductions in these neuropeptides from the pre-synaptic AgRP neurons. Thus, we suggest that leptin and insulin, as well as hunger, may also regulate the post-synaptic events (e.g. NMDA/AMPA trafficking) to influence the neural plasticity.

Elevated AgRP neural activity is sufficient to drive sustained feeding even in satiated animals^3,4^. However, recent in vivo recording efforts revealed that AgRP neurons are rapidly inhibited by the sensory detection of food even before feeding begins^55–57^, which raises a question regarding how feeding behavior is sustained in hungry animals. It has been suggested that the rapid drop in AgRP neural activity on meal onset may reflect a termination of the drive to find food, while residual AgRP neural activity may reflect a sustained drive to consume food^57^. Alternatively, it has been shown that a short stimulation (1–30 min) of AgRP neurons or one of their downstream circuits (e.g. AgRP→PVH, AgRP→BNST, and AgRP→LH) can promote sustained eating even after the AgRP neuron stimulation ceases^58^. Similarly, a pre-stimulation of the AgRP→PBN circuit can trigger increased consumption of high palatable food in satiated mice^59^. These sustained feeding behaviors are linked either to a long-lasting potentiation of the rewarding properties of food or to a delayed satiation^59^. Here the LTDs observed at the AgRP-innervated targets, especially those in POMC neurons, in the PVH, BNST, LH, and PBN may provide the neurobiological basis for a sustained drive to eat when AgRP neuron activity is rapidly inhibited upon initiation of feeding.

Fasting activates AgRP neural firing, while feeding suppresses it^25,26,55,56^. Importantly, AgRP neural activity in fasted mice rapidly reduces in response to food cues before satiation^55–57^. Thus, AgRP neurons function as an important sensor for not only nutritional states but also food cues. Since a short stimulation of AgRP neurons can trigger neural plasticity in multiple targeted neurons, including those in the brain regions implicated in cognitive functions, we speculate that the neural plasticity in AgRP-innervated neurons may facilitate animals to learn and remember cues associated with food availability during food seeking. Supporting this possibility, we showed that neonatal ablation of the majority of AgRP neurons impaired food-seeking behavior in the Y-maze test and in the food-baited hole board test. Notably, both these tests required animals to learn and remember the spatial and contextual cues that were associated with food availability. Thus, the impaired performance of AgRP^neoAblation^ mice indicates that AgRP neurons are required for these tasks when food is used as a bait or motivation. In line with our observations, it has been reported that activation of AgRP neurons, either by food deprivation or by ChR2-mediated optogenetic stimulation, enhances mouse’s learning for spatial cues associated with food pellets in the Y-maze test^15^. Interestingly, AgRP^neoAblation^ mice performed normally in the MWM and fear conditioning tests, indicating that the loss of AgRP neurons does not affect spatial/contextual learning and memory when motivation is not related to foods.

The Y-maze conditioning robustly reduced both NMDA and AMPA currents in AgRP-innervated neurons in the PVT. Since NMDA and AMPA reductions were tightly associated with the LTDs observed at the AgRP-originated synapses, these reduced NMDA and AMPA currents likely indicate that the LTDs have developed in vivo in these PVT neurons during the Y-maze conditioning. In addition, AgRP^PVT-Ablation^ mice, which lose a subset of AgRP neurons that project to the PVT, showed impaired food-seeking behavior in the Y-maze test and in the food-baited hole board test. It is worth noting that AgRP neurons do not have collateral projections^11^. Therefore, ablation of PVT-projecting AgRP subset should not affect AgRP projections to other brain regions. Yet, AgRP^PVT-Ablation^ mice largely recapitulated phenotypes observed in AgRP^neoAblation^ mice with the majority of AgRP neurons ablated. Thus, we suggest that the AgRP→PVT projections are necessary to facilitate food-seeking behavior. This possibility is further supported by the observation that optogenetic inhibition of the AgRP→PVT projections impaired animals’ performance in the Y-maze test. However, we found that optogenetic activation of the AgRP→PVT projections, when coupled to conditioning sessions in mice fed ad libitum, failed to enhance animals’ performance in the Y-maze test, indicating that the AgRP→PVT projections alone are not sufficient for food seeking and other AgRP-originated circuits (e.g. those projecting to POMC neurons, the CeA or the BNST) may also contribute to this function. These possibilities remain to be examined.

In summary, our studies revealed that the hunger-sensory neurons, AgRP neurons, trigger the heterosynaptic LTDs in multiple downstream targeted neurons, and this neural plasticity in many regions can be strengthened by hunger but dampened by satiety signals. In particular, the neural plasticity in AgRP-innervated PVT neurons can be induced by a food-seeking task, and loss of PVT-projecting AgRP neurons or inhibition of the AgRP→PVT projections impairs animals’ ability to associate cues with food availability during food seeking. In addition, the LTDs triggered by AgRP neuron activation may also facilitate sustained feeding. Thus, an interesting scenario that one single orexigenic neural node (AgRP neurons) exerts long-lasting inhibitory effects on various anorexigenic neural populations may reflect a conserved mechanism to ensure sufficient nutrient intake and survival during the evolution which has been dominated by periods with food scarcity.

## Materials and methods

### Mice

We crossed NPY-GFP^60^, POMC-CreERT2^34^, and Rosa26-LSL-tdTOMATO^61^ mouse alleles to generate NPY-GFP/POMC-CreERT2/Rosa26-LSL-tdTOMATO mice. Tamoxifen injection in these mice (0.2 g/kg, i.p., at 8 weeks of age) induced Cre activity and therefore led to the expression of tdTOMATO selectively in mature POMC neurons^62^; adjacent AgRP neurons were labelled by GFP. These mice were used for double-patch recordings of the AgRP→POMC synapse as described below.

We crossed AgRP-IRES-Cre mice^10^ to Rosa26-LSL-tdTOMATO mice to generate AgRP-IRES-Cre/Rosa26-LSL-tdTOMATO mice. These mice were used for WGA-CRACM recordings of AgRP-innervated distant targets as described below. Some AgRP-IRES-Cre mice and their wild-type littermates were used for optogenetic studies as described below.

We crossed AgRP^DTR/+^ mice^6^ with C57/Bl6j mice to generate AgRP^DTR/+^ mice and their wild-type littermates. To generate AgRP^neoAblation^ mice, AgRP^DTR/+^ mice were injected with diphtheria toxin (DT, 50 ng/g, s.c.; #D0564, Sigma-Aldrich) during the first week after birth^6,42^. Two types of controls were included: AgRP^DTR/+^ mice that received saline injections and wild-type littermates that received DT injections. These mice were used for feeding and behavioral studies as described below.

To generate AgRP^PVT-Ablation^ mice, AgRP^DTR/+^ mice (16 weeks of age) received stereotaxic injections of DT (1 ng in 200 nl saline) into the PVT (at the middle line, 1.34 mm posterior and 3.13 mm ventral to the Bregma). Two types of controls were included: AgRP^DTR/+^ mice that received saline injections into the PVT and wild-type littermates that received DT injections into the PVT. AAV-GFP was mixed with DT or saline solution to enable post hoc validation of accurate PVT injections as evaluated by GFP signals in the PVT. After the completion of behavioral studies (described below), all mice were perfused with 10% formalin and brain sections were cut at 25 μm (five series). These brain sections were subjected to immunofluorescence staining for AgRP with rabbit anti-AgRP antibody (1:200, H-003-57, Phoenix Pharmaceuticals) overnight, followed by the donkey anti-rabbit AlexaFluor 594 (1:1000, A21207, Life Technologies) for 1.5 h. Slides were coverslipped and analyzed using a Leica DM5500 fluorescence microscope. The lack of AgRP immunoreactivity in the PVT but the presence in the other AgRP target regions (e.g. PVH) was used to validate selective ablation of PVT-projecting AgRP neurons. As an additional validation, we crossed AgRP^DTR/+^ mice with NPY-GFP mice to generate AgRP^DTR/+^/NPY-GFP mice, which were also subjected to PVT DT or saline injections; post hoc quantifications of GFP-positive neurons in the ARH were performed to validate selective deletion of AgRP subset. Behavioral data only from those mice with accurate ablation were included in the analyses.

Care of all animals and procedures were approved by Baylor College of Medicine Institutional Animal Care and Use Committees. Mice were housed in a temperature controlled at 22–24 °C using a 12-h light, 12-h dark cycle. The mice were fed regular chow (6.5% fat, #2920, Harlan-Teklad, Madison, WI). Water was provided ad libitum.

### Food intake

A cohort of AgRP^neoAblation^ mice and their controls (12–16 weeks of age) were acclimated into the BioDAQ feeding chambers (Research Diets, Inc), supplied with the regular chow diet. These mice were subjected to a 3-day feeding protocol, comprising of 24 h ad libitum feeding, 24 h fasting, and 24 h refeeding. The removal and re-prevision of the diet took place at 6 pm (the onset of dark cycle) of day 2 and day 3, respectively. Food intake was monitored by the BioDAQ system.

A cohort of AgRP^PVT-Ablation^ mice and their controls (4–5 months of age) were acclimated to singly housing supplied with the regular chow diet. These mice were subjected to the 3-day feeding protocol, comprising of 24 h ad libitum feeding, 24 h fasting, and 24 h refeeding. The removal and re-prevision of the diet took place at 6 pm (the onset of dark cycle) of day 2 and day 3, respectively. Food intake during day 1 and day 3 was measured.

### Y-maze test

AgRP^neoAblation^ mice, AgRP^PVT-Ablation^ mice, and their controls were used in the Y-maze test. One arm was deemed the “start arm”, where animals would begin each trial. The other two “choice” arms, coupled to different contexture cues on the floor and walls, contained 50 ml conical tube caps toward the distal end, which were either empty or contained a chow pellet. All mice were acclimated to the pellets and caps beforehand by putting the pellets and caps inside the cage before conditioning to prevent neophobia. Prior to conditioning, all animals were fasted overnight to perform a pre-test. All animals received a 15-min habituation period in the maze before the start of the experiment. Mice were placed in the “start arm” and scored for distribution into one of the two “choice” arms in the absence of any food reward for ten trials. Then, all animals were food restricted and maintained at 85% body weight to motivate exploration of the maze and increase the salience of a food reward, followed by 5-day conditioning sessions. During conditioning, each animal received a forced-choice trial toward the unpaired arm (empty cap) and a forced-choice trial toward the paired arm (baited cap) daily for five consecutive days for a total of 10 sets of conditioning trials. For each set of trials, animals were conditioned to each side for 5 min. After each trial, the maze was cleaned of any excrement and odorant cues. On the test day, all mice (after an overnight fast) received ten free-choice trials with access to both arms, both of which were devoid of food (empty caps). The trial ended as soon as the animal’s center-point crossed fully into one of the arms, signifying a choice to enter one of the two arms, and the distribution was recorded. The number of entries to the food-coupled arm was recorded.

In addition, to examine the effects of inhibition of the AgRP→PVT circuit on animals’ performance in the Y-maze test, we stereotaxically injected AAV-EF1α-DIO-eNpHR3.0-EYFP (3 × 10^12^ VM/ml, 200 nl) into the ARH (bilateral, +/–0.3 mm lateral, 1.60 mm posterior, 5.90 mm ventral to the Bregma) of AgRP-IRES-Cre mice and their wild-type littermates, and implanted an optic fiber to target the PVT (ML 0 mm, AP –1.34 mm, DV –2.8 mm). These mice were subjected to the Y-maze pre-test (after an overnight fasting), conditioning (food restricted to lose 15% body weight), and test (after an overnight fasting), similarly as described above, during which yellow light pulses (589 nm, 10 ms/pulse, 7 mW, 3 Hz) were applied to the PVT through the optic fiber. Further, to examine the effects of activation of the AgRP→PVT circuit on the animals’ performance in the Y-maze test, we stereotaxically injected AAV-EF1α-DIO-hChR2(H134R)-EYFP (6.2 × 10^12^ VP/ml, 200 nl) into the ARH of AgRP-IRES-Cre mice and their wild-type littermates, and implanted an optic fiber to target the PVT. These mice were subjected to the Y-maze pre-test (after an overnight fasting), conditioning (fed ad libitum), and test (after an overnight fasting), similarly as described above, during which blue light pulses (473 nm, 10 ms/pulse, 7 mW, 0.3 Hz) were applied to the PVT through the optic fiber. After the completion of the behavioral studies, all mice were perfused and the brain sections were mounted to validate the expression of eNpHR3.0-EYFP or hChR2(H134R)-EYFP. Slides were coverslipped and analyzed using a Leica DM5500 fluorescence microscope. AgRP neuron cell bodies in the ARH and fibers/boutons in the PVT and PVH were visualized by the expression of EYFP. Behavioral data only from those mice with accurate ablation were included in the analyses.

### Food-baited hole board test

The food-baited hole board test was conducted in a clear Plexiglas open-field arena (40 cm × 40 cm × 30 cm). The hole board with 16 equidistant holes baited with high palatable food pellets was placed in the open-field chamber. Food baits from four of these holes were accessible to mice while food baits in the other 12 holes were inaccessible due to wire lids. On days 1–4, mice that have been fed ad libitum were subjected to a 10-min training session every day at around 12–2 pm. In each training session, a mouse was put at the same starting position to explore the hole board. After each training session, the hole board floor insert was cleaned with alcohol to homogenize potential olfactory traces. Mice were fasted overnight and then subjected to a 10-min testing session on day 5. The nose pokes to the four holes with accessible food baits with a duration longer than 1 s were counted as correct pokes. The nose pokes that lasted less than 1 s and the nose pokes to holes with inaccessible food baits were counted as incorrect pokes.

### Morris water maze

The MWM test was done as we described before^63^ with minor modifications under dim light. All the behaviors in the water maze were recorded by a video camera. Briefly, mice were placed on the transparent rescue platform submerged under the painted water (0.5–1 cm), and let to stand on the platform for 10 s on the first training day. After that, mice were gently placed into the water (25 ^o^C ± 20 ^o^C) facing the wall of the pool and were allowed to explore the pool for 1 min. The escape latency time was recorded when mice stood on the rescue platform. Mice were guided to the rescue platform if they failed to find it during the training with the escape latency marked as 60 s. Mice were allowed to sit on the platform for 10 s, and then were entrained to the water maze from a different start position with the same procedure. After four training trials, mice were dried by a paper towel and returned to their home cages. Twenty-four hours later, mice were trained again following the same procedure without the session of the habituation on the rescue platform. Totally, mice were trained consecutively for 5 days. The mouse behaviors were analyzed by the Noldus EthoVision XT. The escape latency for each day was the average of four trials in the same day of each mouse and served as an independent measurement of spatial learning and memory.

### Fear conditioning

The fear conditioning test was done followed by the previously published method^64^ with minor modifications. Mice were placed in a separated preparation room. On the training day, mice were put into a sound-proof operant chamber and were allowed to freely explore the whole chamber for 2 min. After this acclimation, mice were administered two series of a 30 s, 80 dB white noise tone each, followed immediately by a 2 s, 0.7 mA foot shock. Twenty-four hours later, the mice were tested for contextual memory by placing them in the same sound-proof operant chamber without any additional stimulus for 5 min. One hour later, the cue memory was tested by placing the mice in a novel context for 3 min (pre-tone), followed by a 3 min, 80 dB white noise tone. All behavioral data were analyzed by experimenters who were blinded to the treatment groups.

### Double-patch recording of the AgRP→POMC synapse

Double-patch whole-cell clamp recordings were performed in tdTOMATO-labelled POMC neurons and GFP-labelled AgRP neurons in the ARH-containing hypothalamic slices from NPY-GFP/POMC-CreERT2/Rosa26-LSL-tdTOMATO mice. Briefly, mice (9–12 weeks of age) were deeply anesthetized with isoflurane in the early morning with or without an overnight fast and transcardially perfused with a modified ice-cold sucrose-based cutting solution containing (in mM) 10 NaCl, 25 NaHCO_3_, 195 sucrose, 5 glucose, 2.5 KCl, 1.25 NaH_2_PO_4_, 2 Na pyruvate, 0.5 CaCl_2_, 7 MgCl_2_ bubbled continuously with 95% O_2_ and 5% CO_2_. Three brain slices containing the ARH were obtained for each animal (Bregma –2.06 mm to –1.46 mm; Interaural 1.74 mm to 2.34 mm), and recordings were made at levels throughout this brain region. The slices were recovered for 1 h at 34 °C and then maintained at room temperature in artificial cerebrospinal fluid (aCSF, pH 7.3) containing 126 mM NaCl, 2.5 mM KCl, 2.4 mM CaCl_2_, 1.2 mM NaH_2_PO_4_, 1.2 mM MgCl_2_, 11.1 mM glucose, and 21.4 mM NaHCO_3_ saturated with 95% O_2_ and 5% CO_2_ before recording.

Slices were transferred to a recording chamber and allowed to equilibrate for at least 10 min before the recording. The slices were superfused at 34 °C in oxygenated aCSF at a flow rate of 1.8–2 ml/min. GFP or tdTOMATO-labelled neurons in the ARH were visualized using epifluorescence and IR-DIC imaging on an upright microscope (Eclipse FN-1, Nikon) equipped with a moveable stage (MP-285, Sutter Instrument). Patch pipettes with resistances of 3–5 MΩ were filled with an intracellular solution (pH 7.3) containing 128 mM K-gluconate, 10 mM KCl, 10 mM HEPES, 0.1 mM EGTA, 2 mM MgCl_2_, 0.05 mM Na-GTP, and 0.05 mM Mg-ATP. Recordings were made using a MultiClamp 700B amplifier (Axon Instrument), sampled using Digidata 1440 A, and analyzed offline with pClamp 10.3 software (Axon Instruments). Series resistance was monitored during the recording, and the values were generally <10 MΩ and were not compensated. All the data we used for analysis did not have series resistance changed over 10 MΩ before and after the recording. The liquid junction potential was +12.5 mV and was corrected after the experiment. Data were excluded if the series resistance increased dramatically during the experiment or without overshoot for the action potential. Currents were amplified, filtered at 1 kHz, and digitized at 20 kHz. Current clamp was engaged to test neural firing frequency and RM. A double patch was used to record one AgRP neuron and one adjacent POMC neuron at the same time. In order to determine if the recorded AgRP neuron synapsed onto the recorded POMC neuron, we injected a negative current (–40 pA for 3–4 min) into the AgRP neuron and monitored the firing activity of POMC neurons under the current-clamp mode. In some recorded pairs, we did not observe any obvious changes in the POMC neuron activity upon inhibition of the AgRP neuron, which indicated that the AgRP neuron did not likely synapse on the recorded POMC neuron. In some recorded pairs, we observed instant elevations of firing activity in the POMC neuron upon AgRP neuron inhibition, which instantly recovered upon the completion of the AgRP neuron inhibition, suggesting that the AgRP neuron synapsed on the recorded POMC neuron. To further confirm the AgRP→POMC synaptic connectivity, we used the current clamp model to apply a strong positive current (100 pA, 10 ms) to the AgRP neuron cell body and dendrite to stimulate GABA release from the fiber terminal, and used the voltage-clamp mode to measure evoked IPSCs in the POMC neuron. The eIPSCs in POMC neurons were further examined in the presence of bath perfusion of 50 µM bicuculline (GABA_A_ receptor antagonist) to confirm the GABAergic nature; the eIPSCs in POMC neurons were also examined in the presence of bath perfusion of 1 µM TTX and 400 µM 4-AP to further confirm the monosynaptic connection between the upstream AgRP neuron and the downstream POMC neuron.

After confirming the AgRP→POMC synapse, we switched the bath solution to Mg^2+^ free aCSF. We then injected a train of positive currents (100 pA, 10 ms pulses at 0.3 Hz for 10 min) into the pre-synaptic AgRP neuron, and sEPSC in the post-synaptic POMC neuron was recorded for 20 min (0–20 min) prior to the train stimulation and for 60 min (30–90 min) afterward. The sEPSCs in POMC neurons were recorded in whole-cell voltage-clamp mode in the presence of 50 µM bicuculline, by holding the membrane potential at Vh = –60 mV. The pipette solution containing: 125 mM CsCH_3_SO_3_; 10 mM CsCl; 5 mM NaCl; 2 mM MgCl_2_; 1 mM EGTA; 10 mM HEPES; 5 mM (Mg)ATP; 0.3 mM (Na)GTP (pH 7.3 with NaOH). The amplitude of sEPSCs was calculated for an average in a 1-min bin. In some experiments, the slices were incubated with leptin (300 nM), insulin (300 nM), CGP52432 (50 µM; a GABA_B_ receptor inhibitor)^65^, MTII (100 µM, a MC3/4 R agonist)^66^, or a cocktail of BVD10 (50 µM; a Y1 antagonist)^67^, JNJ5207787 (100 µM; a Y2 antagonist)^68^, and NPY5RA972 (100 µM; a Y5 antagonist)^69^.

The NMDAR and AMPAR sEPSC currents were recorded similarly as reported by others^27^. Briefly, neurons were recorded in the presence of 50 µM bicuculline in whole-cell voltage-clamp mode by holding the membrane potential at Vh = −60 mV. The pipette solution contained 125 mM CsCH_3_SO_3_, 10 mM CsCl, 5 mM NaCl, 2 mM MgCl_2_, 1 mM EGTA, 10 mM HEPES, 5 mM (Mg)ATP, and 0.3 mM (Na)_2_GTP (pH 7.3 with NaOH). A baseline of average sEPSC total current amplitude was recorded and analyzed. NMDAR sEPSC amplitude was calculated by subtracting the average sEPSC total current amplitude in the presence of 50 μM D-AP5 from that recorded in its absence. AMPAR sEPSC amplitude was then calculated by subtracting the background current amplitude (recorded in the presence of 50 μM D-AP5 and 30 μM CNQX) from that recorded in the presence of D-AP5 only.

### WGA-CRACM recording of AgRP-originated distant synapses

AgRP-IRES-Cre/Rosa26-LSL-tdTOMATO mice (8–12 weeks of age) received stereotaxic injections of AAV8-EF1α-DIO-hChR2(H134R)-EYFP (100 nl, 6.2 × 10^12^ VP/ml) and Ad-iN/WED (100 nl, 6.1 × 10^12^ VP/ml)^39^ viruses into both sides of the ARH (+/–0.3 mm lateral, 1.60 mm posterior, 5.90 mm ventral to the Bregma). Four weeks later, mice were deeply anesthetized with isoflurane in the early morning with or without an overnight fast and transcardially perfused as described above. We used the whole-cell patch to record WGA-GFP-labelled neurons in various brain regions, including the PVH, PVT, CeA, MeA, PBN, and BNST. For all recordings, we first used the CRACM protocol^40^ to confirm the synaptic connectivity between the ChR2-labelled AgRP terminals and WGA-GFP-labelled neurons. Thus, a single blue light pulse (473 nm, 40 mW, 10 ms) was applied to the ChR2-labelled fibers/terminals, and we monitored evoked IPSCs in WGA-GFP-labelled neurons under the voltage-clamp mode. The evoked IPSCs were further examined in the presence of 50 µM bicuculline (GABA_A_ receptor antagonist), or in the presence of 400 µM 4-AP and 1 µM TTX to further confirm the monosynaptic connection between the upstream AgRP neurons and the downstream WGA-GFP-labelled neurons.

Then a train of blue light pulses (473 nm, 40 mW, 0.3 Hz pulses for 10 min) was applied to the ChR2-labelled fibers/terminals, and sEPSC in the WGA-GFP-labelled neuron were recorded for 20 min (0–20 min) prior to the train photostimulation and for 30 min (30–50 min) afterward, as described above. Similarly, NMDAR and AMPAR sEPSC were measured before and after the photostimulation.

### Y-maze-induced neural plasticity at AgRP-innervated distant targets

As described above, AgRP-IRES-Cre/Rosa26-LSL-tdTOMATO mice (8–12 weeks of age) received stereotaxic injections of AAV-EF1α-DIO-hChR2(H134R)-EYFP (100 nl, 6.2 × 10^12^ VP/ml) and Ad-iN/WED (100 nl, 6.1 × 10^12^ VP/ml) viruses into both sides of the ARH. Four weeks later, mice were divided into two weight-matched groups. One group was subjected to the Y-maze conditioning for 4 days. On each day, mice were fasted overnight, subjected to the Y-maze conditioning in the morning, and then refed for the rest of the day cycle. After the last conditioning (on day 4), mice were allowed refeeding ad libitum for 24 h. Twenty-four hours after the last conditioning, these mice were euthanized and used for electrophysiological recordings. The other group of weight-matched naive AgRP-IRES-Cre/Rosa26-LSL-tdTOMATO mice that underwent the same viral infections but were not trained in the Y-maze was used as controls. We first recorded tdTOMATO-labelled AgRP neurons in the ARH under the current clamp to confirm that the firing frequency and RM of AgRP neurons were comparable between trained and control mice. We then targeted the WGA-labelled neurons in the CeA, the BNST, and the PVT. As described above, we first used the CRACM protocol to detect time-locked IPSCs in WGA-labelled neurons evoked by blue light pulses shed on the ChR2-expression AgRP terminals. We then measured NMDAR and AMPAR sEPSC in these identified AgRP-innervated neurons, as described above.

### Real-time qPCR analyses

To validate the loss of AgRP neurons from AgRP^neoAblation^ mice, a cohort of AgRP^neoAblation^ mice and their controls (16 weeks of age) were euthanized and the hypothalamus was quickly isolated. Total RNA was isolated using TRIzol Reagent (Invitrogen) according to the manufacturer’s protocol and reverse transcription reactions were performed from 2 μg of total RNA using High-Capacity cDNA Reverse Transcription Kits (Invitrogen). cDNA samples were amplified on a CFX384 Real-Time System (Bio-Rad) using SsoADV SYBR Green Supermix (Bio-Rad). Correct melting temperatures for all products were verified after amplification. Results were normalized against the expression of house-keeping gene-b-actin. Primer sequences are listed in Supplementary Table 1.

### Statistics

The minimal sample size was pre-determined by the nature of the experiments. For most of the physiological readouts (food intake, behaviors, etc.), at least six mice per group were included. For histology studies, three mice were included in each group. For electrophysiological studies, at least four neurons in each genotype or condition were included. The data are presented as mean ± SEM or as individual data points. Statistical analyses were performed using GraphPad Prism to evaluate normal distribution and variations within and among groups. Methods of statistical analyses were chosen based on the design of each experiment and are indicated in figure legends. *P* < 0.05 was considered to be statistically significant.

### Study approval

Care of all animals and procedures were approved by the Baylor College of Medicine Institutional Animal Care and Use Committee.

## Supplementary information

Supplementary Figures and Tables
