# Supplementary material for: AgRP neurons trigger long-term potentiation and facilitate food seeking
Source: Transl Psychiatry. 2021 Jan 5;11:11. doi: 10.1038/s41398-020-01161-1 (PMC7791100; doi:10.1038/s41398-020-01161-1)
Supplement: Supplementary file 1 — Supplementary Figures and Tables [file 41398_2020_1161_MOESM1_ESM.docx]

**Supplementary Information**

**Supplementary Figures**

**
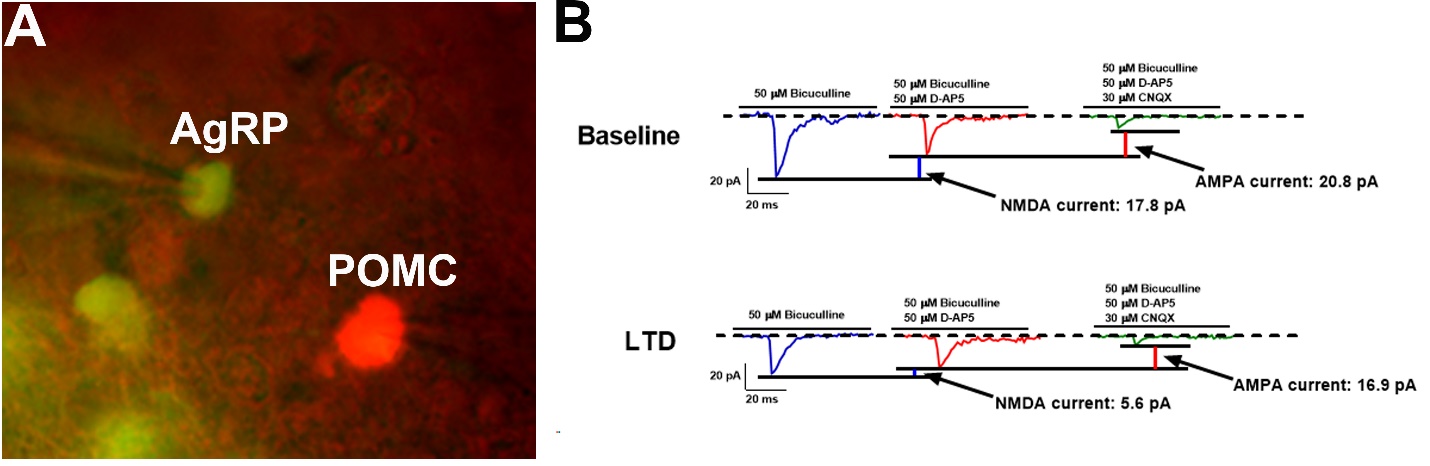
**

**Figure S1 (Related to Figure 1 and 2).** **sEPSC LTD at the AgRP🡪POMC synapse.** (A) A representative image showing a live POMC neuron (red) and a live AgRP neuron (green) under double patch recording. (B) Representative sEPSC traces recorded from POMC neurons (from fed mice) at the baseline or LTD condition in the presence of various inhibitors. The calculations of NMDA and AMPA currents were indicated by arrows.


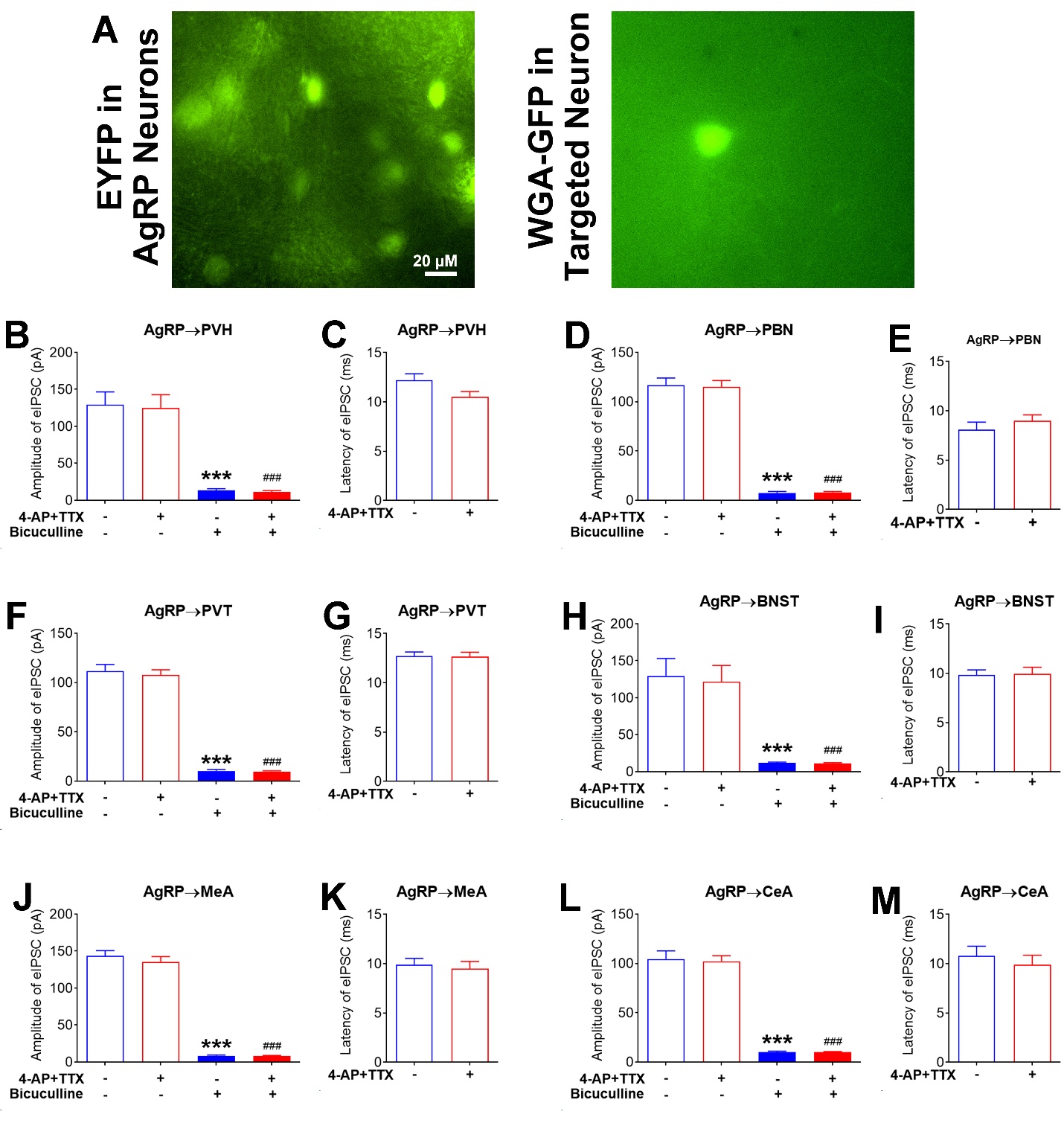


**Figure S2 (Related to Figure 3).** **AgRP neurons trigger sEPSC LTDs in distant synaptic targets.** (A) Representative images showing live ChR2-expressing AgRP neurons (left) and a live WGA-labelled neuron (right) in distant targeted regions. (B, D, F, H, J, L) Amplitude of blue light-evoked IPSC recorded in WGA-labelled neurons in the PVH (B), PBN (D), PVT (F), BNST (H), MeA (J) and CeA (L) in the presence or absence of 4-AP and TTX, and/or bicuculline. Data are mean±SEM. N=5 or 6 per group. ***, P<0.001 vs the first group; ###, P<0.001 vs. the second group in one way ANOVA analyses followed by post hoc Tukey’s tests. (C, E, G, I, K, M) Latency of blue light-evoked IPSC recorded in WGA-labelled neurons in the PVH (C), PBN (E), PVT (G), BNST (I), MeA (K) and CeA (M) in the presence or absence of 4-AP and TTX. Data are mean±SEM. N=5 or 6 per group. No significance in two-tailed paired t-tests.


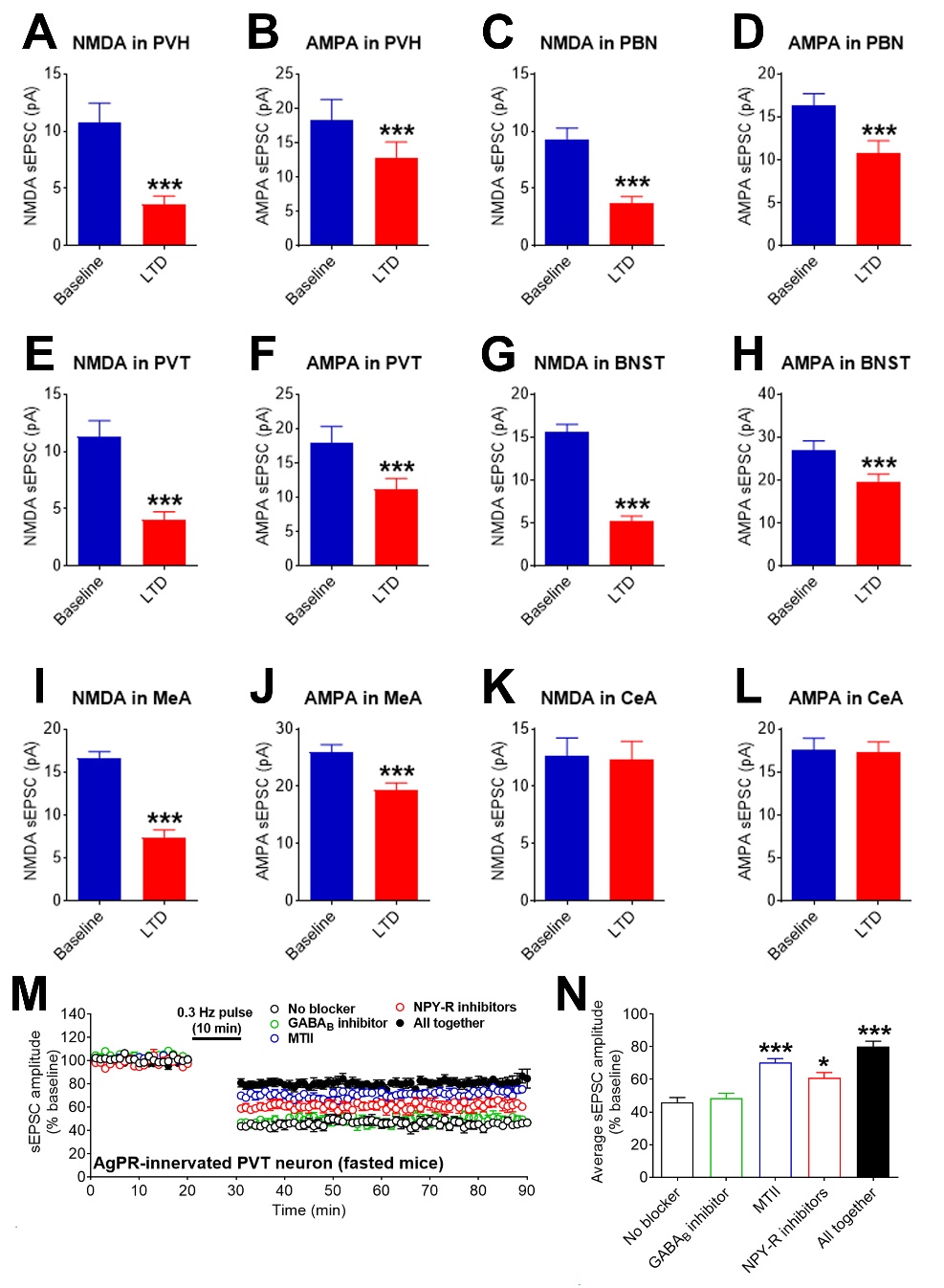


**Figure S3 (Related to Figure 3).** **AgRP neurons trigger sEPSC LTDs in distant synaptic targets.** (A-B) Amplitude of NMDA (A) and AMPA (B) sEPSC in AgRP-innervated neurons in the PVH before and after LTD induction. (C-D) Amplitude of NMDA (C) and AMPA (D) sEPSC in AgRP-innervated neurons in the PBN before and after LTD induction. (E-F) Amplitude of NMDA (E) and AMPA (F) sEPSC in AgRP-innervated neurons in the PVT before and after LTD induction. (G-H) Amplitude of NMDA (G) and AMPA (H) sEPSC in AgRP-innervated neurons in the BNST before and after LTD induction. (I-J) Amplitude of NMDA (I) and AMPA (J) sEPSC in AgRP-innervated neurons in the MeA before and after LTD induction. (K-L) Amplitude of NMDA (K) and AMPA (L) sEPSC in AgRP-innervated neurons in the CeA before and after LTD induction. Data are mean±SEM. N=5 or 6 per group. ***, P<0.001 vs the baseline in two-tailed paired t-tests. (M) Temporal changes in sEPSC amplitudes in AgRP-innervated PVT neurons POMC neurons before and after the 10-min AgRP neuron stimulation (from fasted mice) after the brain slices being pre-incubated with various blockers. (N) The average sEPSC amplitude after AgRP stimulation in (G). Data are mean±SEM. N=6 per group. *, P<0.05 and ***, P<0.001 vs. the no blocker group in one way ANOVA analyses followed by post hoc Tukey’s tests.


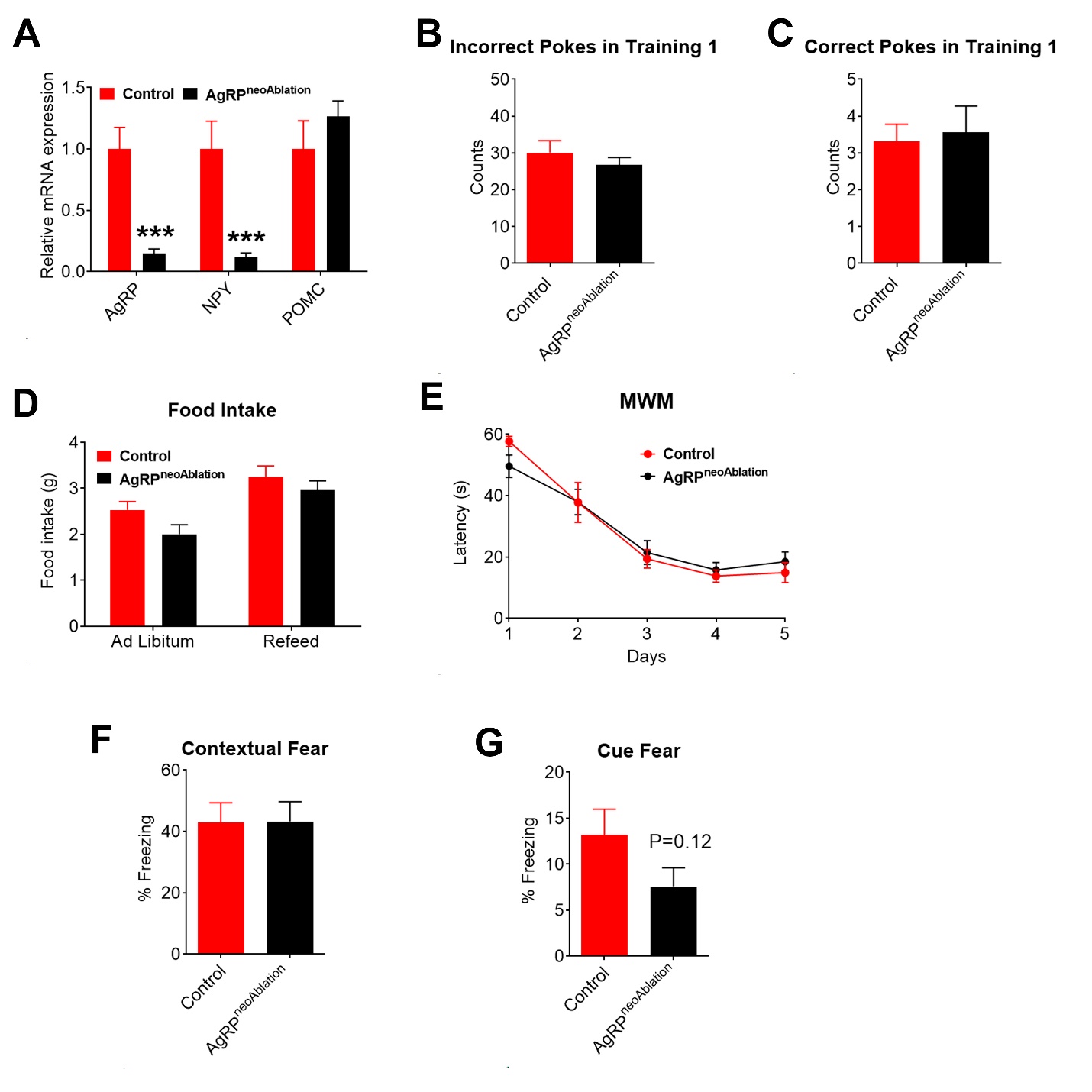


**Figure S4 (Related to Figure 4).** **Loss of AgRP neurons impairs food seeking.** (A) Validation of AgRP^neoAblation^ mice. Real-time RT-PCR quantifications of indicated mRNAs in the ARH from control vs. AgRP^neoAblation^ mice. Data are mean±SEM. N=6 or 8 per group. ***, P<0.001 in two-tailed unpaired t-tests. (B-C) Incorrect (B) and correct pokes (C) of control and AgRP^neoAblation^ mice in the first training session of the food-baited hole board test. Data are mean±SEM. N=16 per group. No significance in two-tailed unpaired t-tests. (D) Daily food intake of control and AgRP^neoAblation^ mice at ad libitum condition or during 24-hr refeeding after a 24-hr fasting. Data are mean±SEM. N=6 or 7 per group. No significance in two-tailed unpaired t-tests. (E) Learning curves of control and AgRP^neoAblation^ mice in the Morris water maze test. Data are mean±SEM. N=6 or 12 per group. No significance in two-way ANOVA or in two-tailed unpaired t-tests for each time point. (F and G) Contextual (F) and cue memory (G) of control and AgRP^neoAblation^ mice in the fear conditioning test. Data are mean±SEM. N=6 or 12 per group. No significance in two-tailed unpaired t-tests.


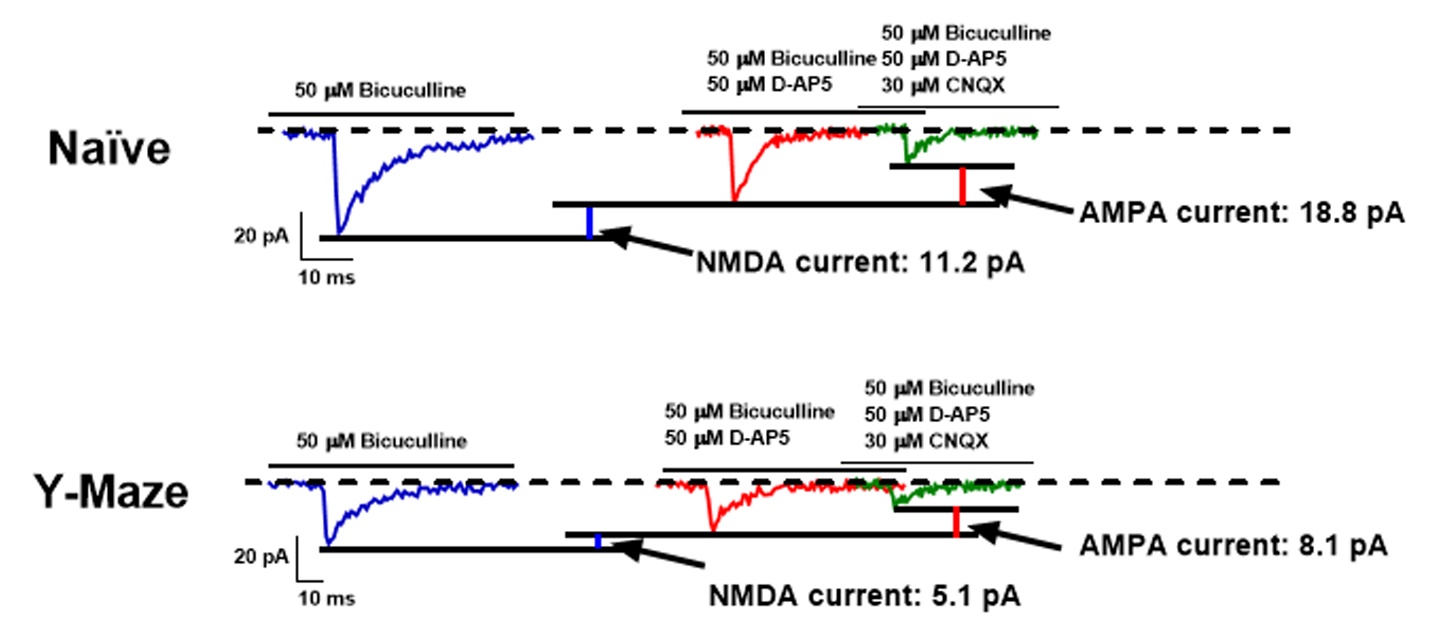


**Figure S5 (related to Figure 5). Y-maze conditioning induced neural plasticity in AgRP-innervated PVT neurons.** Representative sEPSC traces recorded from AgRP-innervated PVT neurons at the baseline or LTD condition in the presence of various inhibitors. The calculations of NMDA and AMPA currents were indicated by arrows.

**
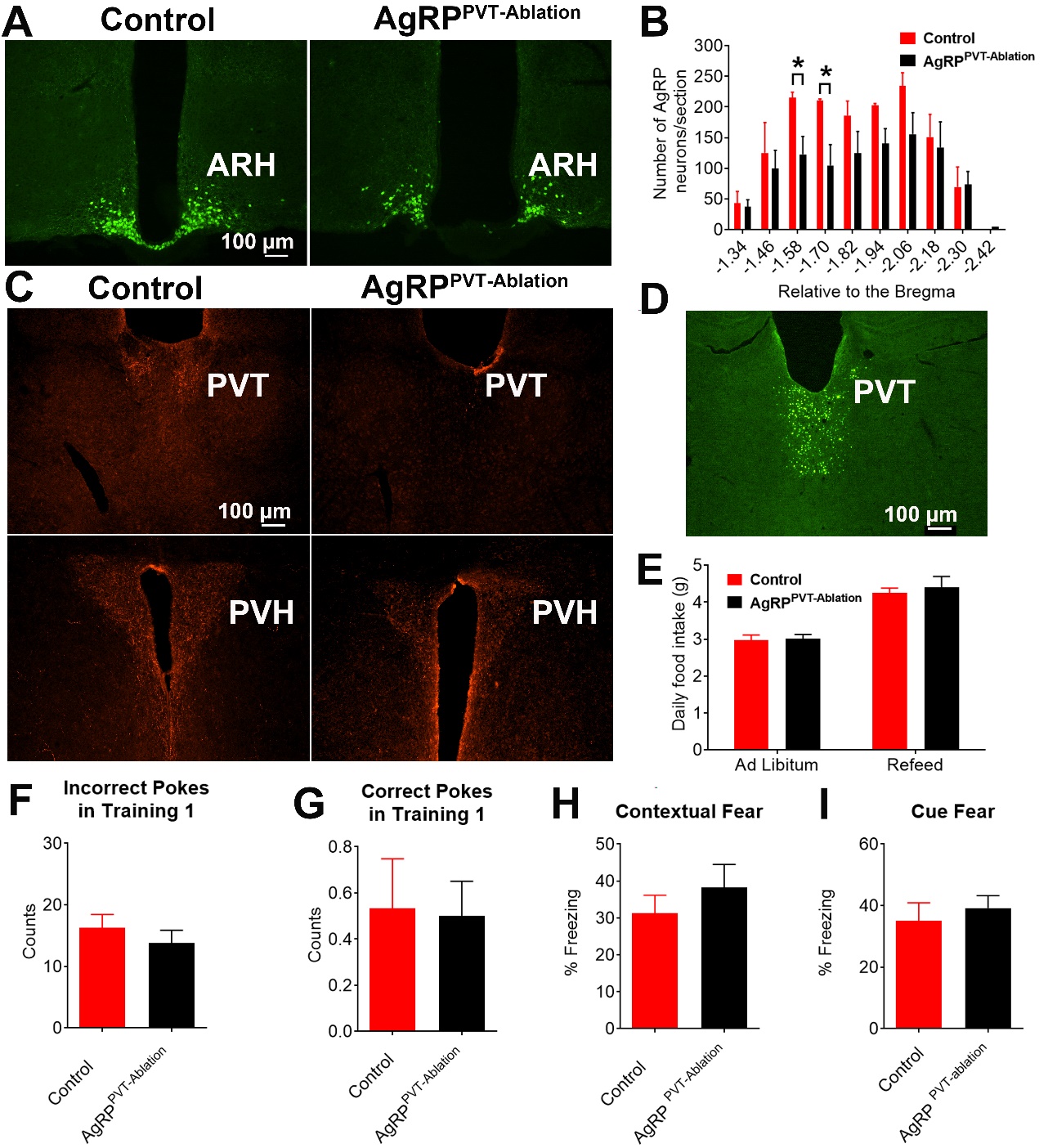
**

**Figure S6 (related to Figure 6). Loss of PVT-projecting AgRP neurons impairs food seeking.** (A) Representative microscopic images showing GFP-labelled AgRP neurons in control and AgRP^VTA-Ablation^ mice. AgRP neurons are labelled by GFP due to the presence of NPY-GFP allele in these mice. Scale bar=100 µm. (B) Quantification of AgRP neuron numbers. Data are mean±SEM. N=3 or 4 per group. *, P<0.05 vs. control group in two-tailed unpaired t-tests. (C) Representative microscopic images showing AgRP-immunoreactive fibers/boutons in the PVT and PVH of control and AgRP^VTA-Ablation^ mice. Scale bar=100 µm. (D) A representative microscopic image showing GFP (carried by AAV-GFP) validating accurate injection in the PVT. Scale bar=100 µm. (E) Daily food intake of control and AgRP^VTA-Ablation^ mice at ad libitum condition or during 24-hr refeeding after a 24-hr fasting. Data are mean±SEM. N=4 or 8 per group. (F-G) Incorrect (F) and correct pokes (G) of control and AgRP^VTA-Ablation^ mice in the first training session of the food-baited hole board test. Data are mean±SEM. N=12 or 15 per group. (H and I) Contextual (H) and cue memory (I) of control and AgRP^VTA-Ablation^ mice in the fear conditioning test. Data are mean±SEM. N=6 or 10 per group.


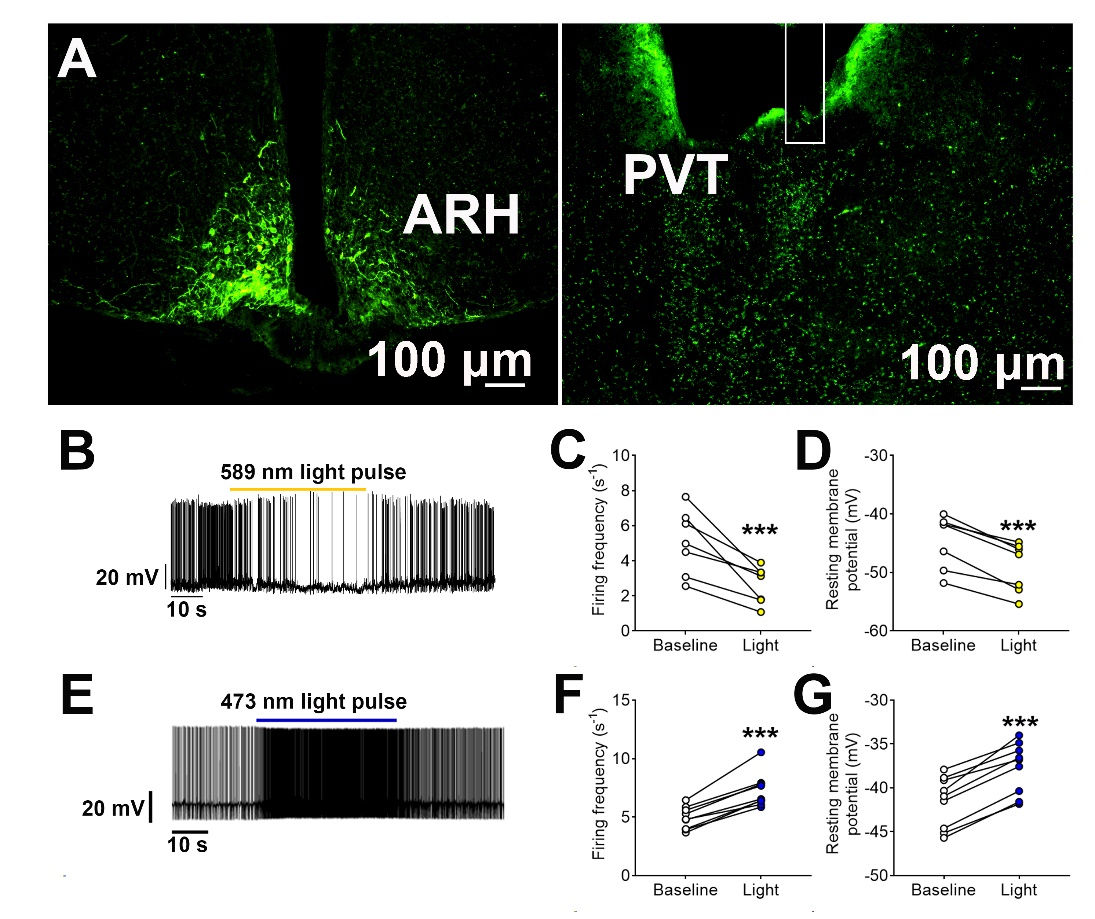


**Figure S7 (related to Figure 6). Inhibition of AgRP🡪PVT projections impairs food seeking.** (A) Representative microscopic images showing eNpHR3.0-EYFP-labelled AgRP cell bodies in the ARH and the fibers/boutons in the PVT. The white box indicates the track of the optic fiber. Scale bars=100 µm. (B) Representative action potential traces recorded from an eNpHR3.0-EYFP-expressing AgRP neuron in response to yellow light stimulation (589 nm). (C-D) Firing frequency (C) and resting membrane potential (D) of eNpHR3.0-EYFP-expressing AgRP neurons in response to yellow light stimulation. ***, P<0.001 in two-tailed paired t-tests. (E) Representative action potential traces recorded from a ChR2-EYFP-expressing AgRP neuron in response to blue light stimulation (473 nm). (F-G) Firing frequency (F) and resting membrane potential (G) of ChR2-EYFP-expressing AgRP neurons in response to blue light stimulation.

**Supplemental Table 1 (related to Figure 3).** Sequences of primers used for qPCR analyses.

| **Gene/protein name** | **Species** | **Primer Sequence** |
| --- | --- | --- |
| POMC | mouse | GAGGCCACTGAACATCTTTGTC |
|  |  | GCAGAGGCAAACAAGATTGG |
| NPY | mouse | CTACTCCGCTCTGCGACACT |
|  |  | AGTGTCTCAGGGCTGGATCTC |
| AgRP | mouse | CGGCCACGAACCTCTGTAG |
|  |  | CTCATCCCCTGCCTTTGC |
| b-actin | mouse | ATGGAGGGGAATACAGCCC |
|  |  | TTCTTTGCAGCTCCTTCGTT |

**KEY RESOURCES**

| **REAGENT or RESOURCE** | **SOURCE** | **IDENTIFIER** |
| --- | --- | --- |
| **Chemicals, Peptides, and Recombinant Proteins** |  |  |
| tamoxifen | Sigma | T-5648 |
| leptin | HARBOR-UCLA Research And Education Institute | N/A |
| insulin | Lilly | NDC 0002-8215-01 |
| diptheria toxin | List Biological Laboratories | N/A |
| CNQX | tocris | 1045 |
| AP-5 | tocris | 0106 |
| bicuculline | tocris | 0131 |
| MT-II | tocris | 2566 |
| CGP52432 | tocris | 1246 |
| BVD10 | tocris | 2177 |
| JNJ5207787 | tocris | 4018 |
| NPY5RA972 | tocris | 3677 |
| **Antibodies** |  |  |
| Rabbit anti-AgRP antibody | Phoenix Pharmaceuticals | H-003-57 |
| Donkey anti-rabbit AlexaFluor 594 | Life Technologies | A21207 |
| **Experimental Models: Organisms/Strains** |  |  |
| Mouse: AgRP^DTR/+^ | Luquet S, et al. 2005 | N/A |
| Mouse: Pomc-CreERT2 | Berglund, et al. 2013 | N/A |
| Mouse: AgRP-IRES-Cre | Jackson Laboratory | 012899 |
| Mouse: Rosa26-LSL-tdTOMATO | Jackson Laboratory | 007909 |
| Mouse: NPY-GFP | Jackson Laboratory | 006417 |
| **Recombinant DNA** |  |  |
| Ad-iN/WED | Leinninger, et al. 2011 | N/A |
| AAV8-EF1α-DIO-hChR2(H134R)-EYFP | UNC Gene Therapy Center | AV4378G |
| AAV- EF1α-DIO-eNpHR3.0-EYFP | UNC Gene Therapy Center | AV4846C |
| AAV8-hSyn-GFP | UNC Gene Therapy Center | N/A |
